# Supplementary material for: Protocol for a systematic review and meta-analysis of the diagnostic test accuracy of host and HPV DNA methylation in cervical cancer screening and management
Source: BMJ Open. 2023 Jun 5;13(6):e071534. doi: 10.1136/bmjopen-2022-071534 (PMC10254594; doi:10.1136/bmjopen-2022-071534)
Supplement: Supplementary data [file bmjopen-2022-071534supp001.pdf]

FIGURE LEGENDS

Figure 1

NHSCSP HPV primary screening programme. This highlights the role of the triage test (currently cytology within UK) in the cervical screening programme algorithm, for management of women detected as hrHPV positive at primary screening, and onward decision tree for colposcopy referral versus repeat screening in 12 months.

Appendix 1

Search strategy

1. Uterine Cervical Neoplasms/
2. Cervical Intraepithelial Neoplasia/
3. CIN*.mp.
4. exp Uterine Cervical Dysplasia/
5. (cervi* adj5 (cancer* or tumor* or tumour* or malignan* or neoplas* or carcinoma* or adenocarcinoma* or dysplasia or squamous or CIN* or HSIL or LSIL or ASCUS)).mp.
6. 1 or 2 or 3 or 4 or 5
7. exp papillomaviridae/
8. exp Papillomavirus Infections/
9. (hpv* or papilloma*).mp.
10. 7 or 8 or 9
11. ((human or host) adj5 methylation).mp.
12. 10 or 11
13. exp methylation/
14. exp Epigenesis, Genetic/
15. Epigenomics/
16. (methylome* or epigenetic* or epigenome*).mp.
17. (bisulphite* or bisulfite* or pyrosequenc* or methylation* or mass spectrometry or mass-spectrometry or EpiTYPER).mp.
18. (next generation sequencing or NGS or high throughput sequencing or illumina array* or illumina sequenc* or solexa).mp.
19. (microsphere-based suspension array* or microsphere based suspension array* or luminex Cmap or luminex C-map or luminex C map).mp.
20. (suspension adj5 (array* or technology*)).mp.

21. 13 or 14 or 15 or 16 or 17 or 18 or 19 or 20
22. exp DNA/
23. DNA.mp.
24. 22 or 23
25. 21 and 24
26. exp DNA Methylation/
27. (DNA adj5 methylation*).mp.
28. 26 or 27
29. 25 or 28
30. 6 and 12 and 29

Appendix 2

QUADAS-2 criteria

Domain	Tailored signalling question	Description
**A) Patient Selection**		
	Was a consecutive or random sample of patients enrolled?	
	Yes	all eligible women consecutively or randomly sampled from a screening population or previous cohort
	No	non-randomly selected (e.g. cases chosen from a biobank)
	Unclear	unclear: not described
	Was a case-control design avoided	
	Yes	not case-control
	No	clear prior selection of cases and controls
	Unclear	unclear: not described
	Were inappropriate exclusions avoided?	
	Yes	yes
	No	inappropriately excluded patients
	Unclear	unclear: not described
	Applicability: Consider whether patients included differ to a hrHPV positive screening population, aged 20-70 years, have comorbid conditions not representative of screening population e.g. other cancer, HIV.	
**B) Index test**		

Were the index test results interpreted without knowledge of the reference standard?

Yes

No

Unclear

If a threshold was used, was it prespecified?

Yes

No

Unclear

Was there an acceptable sample material (LBC, cervical swab, cervical tissue)?

Yes	LBC, cervix swab, tissue
No	vaginal swab or other
Unclear	unclear: not described

Was there an acceptable methylation test giving quantitative results (Pyrosequencing, EpiTYPER, Next generation sequencing, Luminex)?

Yes	well established quantitative technique (Pyro, Epityper, NGS, Luminex-C)
No	binary technique - BS, MSP, MS-HRM, MSRE
Unclear	unclear

Applicability: Are there any concerns regarding the index test used and transference to a screening population of hrHPV positive women?

C) Reference Standard

Is the reference standard likely to correctly classify the target condition? (histology confirmation of grade for CIN and cancer, at least cytology confirmation of grade for normal and ASCUS)?

Yes	histology for all (CIN, Cancer) or mixed cytology and histology as clinically indicated in NHSCSP guidelines

No	cytology for all
Unclear	unclear: not described

Were the reference standard results interpreted without knowledge of the results of the index test?

Yes	all scientists masked to reference test
No	not masked
Unclear	unclear or not described

Applicability: does the target condition in the study represent high-grade CIN or cancer?

D) Flow and timing

Was there an acceptable interval between index test and reference standard – (less than 12 weeks as the condition is unlikely to change during this time)?

Yes	the index test and reference standard were performed on the same patient sample or within a reasonable time frame
No	different samples used or a long delay between index test and reference standard
Unclear	

Did all patients receive the same reference standard?

Yes	either histology or cytology used as reference standard for all samples
No	cytology as reference standard for some samples and histology used for reference standard in other samples e.g. low and high grade
Unclear	unclear: not described

Were all patients included in the analysis?

Yes	withdrawals explained e.g. failed methylation test
No	withdrawals and uninterpretable results not explained

Unclear	unclear: not described
