# Supplementary material for: Protocol for a systematic review and meta-analysis of the diagnostic test accuracy of host and HPV DNA methylation in cervical cancer screening and management
Source: BMJ Open. 2023 Jun 5;13(6):e071534. doi: 10.1136/bmjopen-2022-071534 (PMC10254594; doi:10.1136/bmjopen-2022-071534)
Supplement: Author's manuscript [file bmjopen-2022-071534.draft_revisions.pdf]

BMJ Open

BMJ Open is committed to open peer review. As part of this commitment we make the peer review history of every article we publish publicly available.

When an article is published we post the peer reviewers' comments and the authors' responses online. We also post the versions of the paper that were used during peer review. These are the versions that the peer review comments apply to.

The versions of the paper that follow are the versions that were submitted during the peer review process. They are not the versions of record or the final published versions. They should not be cited or distributed as the published version of this manuscript.

BMJ Open is an open access journal and the full, final, typeset and author-corrected version of record of the manuscript is available on our site with no access controls, subscription charges or pay-per-view fees (http://bmjopen.bmj.com).

If you have any questions on BMJ Open's open peer review process please email info.bmjopen@bmj.com

BMJ Open

Protocol for a Systematic Review and Meta-analysis of the Diagnostic Test Accuracy of host and HPV DNA methylation in cervical cancer screening and management

Journal:	*BMJ Open*
Manuscript ID	bmjopen-2022-071534
Article Type:	Protocol
Date Submitted by the Author:	08-Jan-2023
Complete List of Authors:	Bowden, Sarah J; Imperial College London<br>Ellis, Laura; Imperial College London, Surgery and Cancer<br>Kalliala, Ilkka; Institute of Reproductive and Developmental Biology, Department of Surgery & Cancer, Faculty of Medicine, Imperial College London; University of Helsinki and Helsinki University Hospital, Department of Obstetrics and Gynaecology<br>Paraskevaidi, Maria; Imperial College London, Surgery and Cancer<br>Tighe, Jack; Imperial College London,<br>Kechagias, Konstantinos S.; Imperial College London, Surgery and Cancer<br>Doulgeraki, Triada; Ioannina University Hospital, Department of Obstetrics & Gynaecology<br>Paraskevaidis, Evangelos; University of Ioannina, Department of Obstetrics & Gynaecology<br>Arbyn, Marc ; Belgian Cancer Centre<br>Flanagan, James; Imperial College London<br>Veroniki, Areti; St Michael's Hospital Toronto<br>Kyrgiou, Maria; Imperial College London
Keywords:	Gynaecological oncology < GYNAECOLOGY, Gynaecological oncology < ONCOLOGY, ONCOLOGY, Colposcopy < GYNAECOLOGY, Cancer genetics < GENETICS

SCHOLARONE™
Manuscripts

Protocol for a Systematic Review and Meta-analysis of the Diagnostic Test Accuracy of host and HPV DNA methylation in cervical cancer screening and management

Sarah J Bowden[1*], Laura Burney Ellis[1*], Ilkka Kalliala[2], Maria Paraskevaidi[2], Jack Tighe[3], Konstantinos Kechagias[2], Triada Doulgeraki[4?], Evangelos Paraskevaidis[4], Marc Arbyn[5], James Flanagan[3], Areti A Veroniki[6**], Maria Kyrgiou[7**]

*Joint first authors, **Joint last authors

Corresponding author = Sarah J Bowden.

s.bowden@imperial.ac.uk

laura.ellis@imperial.ac.uk

i.kalliala@imperial.ac.uk

m.paraskevaidi@imperial.ac.uk

jack.tighe18@imperial.ac.uk

konstantinos.kechagias18@imperial.ac.uk

iridoul@gmail.com

vangelispar@hotmail.com

vangelispar@hotmail.com

marc.arbyn@sciensano.be

j.flanagan@imperial.ac.uk

argie.veroniki@gmail.com

m.kyrgiou@imperial.ac.uk

[1]Surgery and Cancer, Imperial College London, London, UK
[2]The Institute of Reproductive and Developmental Biology (IRDB), Surgery and Cancer, Imperial College London, London, UK
[3] Imperial College London, London, UK
[4]Department of Obstetrics & Gynaecology, Ioannina University Hospital, Ioannina, Greece
[5]Unit of Cancer Epidemiology, Belgian Cancer Centre, Sciensano, Brussels, Belgium - Department of Human Structure and Repair, Faculty of Medicine and Health Sciences, University Ghent, Ghent, Belgium
[6]Knowledge Translation Program, Li Ka Shing Knowledge Institute, St Michael's Hospital, Unity Health Toronto, Toronto, ON, Canada - Institute of Health Policy Management and Evaluation, University of Toronto, Toronto, ON, Canada
[7]Surgery and Cancer - West London Gynaecological Cancer Centre, Imperial College London - Queen Charlotte's & Chelsea, Hammersmith Hospital, Imperial NHS Healthcare Trust, London, UK

For peer review only

Keywords

Uterine cervical neoplasms, Cervical Intraepithelial Neoplasia, DNA methylation, Epigenomics, Papillomavirus

Article Summary

Abstract

Introduction

Human papillomavirus (HPV) is necessary but not sufficient for cervical cancer development. During cervical carcinogenesis, methylation levels increase across host and HPV DNA. DNA methylation has been proposed as a test to diagnose cervical intraepithelial neoplasia (CIN); we present a protocol to evaluate the accuracy of methylation markers to detect high-grade CIN and cervical cancer.

Methods and analysis

We will search electronic databases (Medline, Embase, and Cochrane Library), from inception, to identify studies examining DNA methylation as a diagnostic marker for CIN or cervical cancer, in a cervical screening population. The primary outcome will be to assess the diagnostic test accuracy of host and HPV DNA methylation for high-grade CIN; the secondary outcomes will be to examine the accuracy of different methylation cut-off thresholds, and accuracy in high-risk HPV positive women. Our reference standard will be histology. We will perform meta-analyses using Cochrane guidelines for diagnostic test accuracy. We will use the number of true positives, false negatives, true negatives and false positives from individual studies. We will use the bivariate mixed effect model to estimate sensitivity and specificity with 95% confidence intervals; we will employ different bivariate models to estimate sensitivity and specificity at different thresholds if sufficient data per threshold. For insufficient data, the hierarchical summary receiver operating curve model will be used to calculate a summary curve across thresholds. If there is inter- and intra-study variation in thresholds, we will use a linear mixed effects model to calculate the optimum threshold. If few studies are available, we will simplify models by assuming no correlation between sensitivity and specificity and perform univariate, random-effects meta-analysis. We will assess the quality of studies using QUADAS-2 and QUADAS-C.

Ethics and dissemination

Ethical approval is not required. Results will be disseminated to academic beneficiaries, medical practitioners, patients and the public.

For peer review only - http://bmjopen.bmj.com/site/about/guidelines.xhtml

PROSPERO registration number: CRD42022299760

Strengths and Limitations of this Study

1. The results of this meta-analysis will guide targeted further research into specific genes as diagnostic markers for cervical precancer and cancer

2. We will separately analyse diagnostic test accuracy in high-risk HPV positive women, in addition to a general screening population, in order to determine the suitability of DNA methylation as a triage tool as well as a diagnostic test

3. A test with higher diagnostic test accuracy for CIN2 than cytology would have clear clinically significant impact for use as a triage or screening tool

4. Our results will be limited by the quality and quantity of studies in the review

5. Variation in measuring and reporting methylation may lead us to conduct several separate meta-analyses, rather than pooling all data

Word Count 3731

Introduction

Rationale

Cervical cancer continues to be the fourth most common cancer in women globally, resulting in 311,000 deaths in 2018 [1,2]. Human Papillomavirus (HPV) is responsible for 99% cervical cancer; about 14 genotypes of HPV are carcinogenic and classified as high-risk HPV types (hrHPV) [3,4]. Over 70% of women are infected with HPV during their lifetime [5]. In a small number, persistent hrHPV leads to development of precancerous lesions, which can progress to cancer [6].

Cervical precancer (Cervical Intraepithelial Neoplasia (CIN), classified into 3 histological grades CIN1-3), can usually be treated surgically. Where implemented, call-recall cervical screening programmes have dramatically reduced incidence of and mortality from cervical cancer through monitoring and treatment of CIN [7,8]. Such programmes have traditionally used cytology of exfoliated cervical cells as the primary screening test. Cytology testing has limitations as a screening test including a low sensitivity for CIN (53 to 75%) [9-11], high inter- and intra-observer variation and minimal future potential for robotic automation, point-of-care testing, and self-sampling. As a result, much research into alternative tests to cytology has been undertaken in recent years.

For peer review only

HrHPV DNA has been shown to be more sensitive than cytology for detection of precancerous lesions and cancer and is now the primary screening method of choice in many countries, including the UK [12]. Primary hrHPV DNA screening is anticipated to offer 60 to 70% greater protection against invasive cancer compared with cytology-based screening [13]. However, higher sensitivity for CIN is associated with a decreased specificity, resulting in a modest positive predictive value, in part due to the fact that the hrHPV positive prevalence in countries such as the UK is as high as 15%, reaching more than 30% in women aged 30 years or less [14]. A triage test for those found to be positive for hrHPV is needed to improve the specificity of screening and identify only those who are truly at risk of precancerous and cancerous lesions.

Reflex cytology (cytology testing for hrHPV-positive samples on the same specimen), remains the test with the best historic safety data and is part of the national guidelines in many countries, including the UK [15] (Figure 1). However, molecular tests are of increasing interest, particularly human and hrHPV methylation, host methylation, HPV E6 and E7 mRNA testing and over-expression of proteins indicating progression (precancer). Such tests may offer the potential to improve accuracy in diagnosis of progressive CIN, whilst also allowing machine automation, self-sampling, and point-of-care testing in the future.

Methylation is a regulatory chemical process, which occurs at specific methylation sites in DNA, called methylation 5-cytosine-phosphate-guanine-3 (CpG) sites. This causes the switching 'on and off' of specific genes to affect gene expression and determine downstream cellular processes. Methylation has been noted to be an early and frequent event in the development of many cancers, and is commonly observed at high levels in tumour tissues [16].

Studies of DNA methylation so far have focused on the variation in levels of methylation in different grades of CIN and cancer, in both human and HPV genes. In general, an increase in methylation levels have been reported to be associated with increasing grades of cervical disease [17-19]. Diagnostic accuracy studies of human and HPV methylation as markers of high-grade cervical neoplasia have shown promising results [20,21], with good sensitivity and specificity [18,22]. A meta-analysis of HPV genes found HPV L1/L2 pooled sensitivity to be 77% [18], and a systematic review of methylation in host DNA found methylation of FAM19A4 to have a sensitivity between 68.2-86.7%, and specificity 60.6-79.3% [19]. Studies have reported a range of thresholds for cut-off values for DNA methylation as a diagnostic test and examined methylation .

Human and/or hrHPV methylation tests may be able to fulfil the urgent unmet need for a triage test for HPV positive women diagnosed at screening. This systematic review and meta-analysis aims to explore the diagnostic test accuracy of human and/or HPV methylation tests; in women of cervical screening

For peer review only

age, whether a positive methylation test can accurately diagnose High-grade Squamous Intraepithelial Lesion (HSIL)/CIN2+, as defined by histology as the reference standard.

Objectives

Primary Objective

The primary objective is to determine the diagnostic test accuracy of human and viral DNA methylation tests for the detection of CIN grade 2 or worse (CIN2/HSIL+), as defined by histology as a reference standard, in women of cervical screening age.

Secondary objectives

A secondary objective is to evaluate the diagnostic accuracy of human and HPV DNA methylation tests for the detection of CIN2/HSIL+, in different target populations for the use in cervical screening, where studies are available, including:

- Primary screening
- Triage of cytology positive women
- Triage of high-risk HPV positive women
- Post treatment of CIN

Further objectives include evaluation of the diagnostic accuracy of human and HPV DNA methylation for detection of CIN3/HSIL+, or invasive cervical cancer.

We also aim to explore the effect of different cut-off thresholds that are provided for single (or combined) methylation CpG sites (human and/or HPV) in diagnosing CIN2+ or CIN3+.

Also, in the presence of any comparator test in the same study (e.g. cytology or HPV16/18 genotyping), we will analyse relative accuracy of DNA methylation to the alternative test.

Methods and Analysis

This protocol was written in accordance with Preferred Reporting Items for Systematic review and Meta-Analysis Protocols (PRISMA-P) guidelines [23].

Eligibility criteria

Types of participants

For peer review only

Any population of adult women representing a cervical screening setting or referral population will be included, without demographic restrictions, including retrospective biobanks of samples for women recruited from such populations, regardless of hrHPV genotype and sample material. Where data is available, we will separately analyse hrHPV positive women.

Index tests to be considered

All DNA methylation tests will be considered: human DNA methylation tests, hrHPV DNA methylation tests or tests which combine CpG sites from human and hrHPV tests into a single marker.

Although there are several million potential CpG sites in the human genome, we will not limit the review to any particular genes and will report all findings. Within the HPV genome there are 8 genes, containing several hundred CpG sites, which vary according to HPV genotype. All genes will be considered but will be analysed separately according to HPV type. Assays can only be developed for a single genomic region spanning 2-300 base-pairs (bp) however many tests include multiple sites by combining results of different genes into one test score. We will not restrict to either single or multiple site methylation tests but will aim to analyse these separately where possible.

All human DNA and hrHPV DNA methylation technologies will be included: for example we will include (but not limited to) bisulphite clonal sequencing, pyrosequencing, epiTYPER, next generation sequencing (NGS), Luminex, Methylation-Sensitive High Resolution Melting (MS-HRM), Methylation-Specific PCR (MSP) [24]. Both in-house laboratory assays and commercial assays will be included. As many technologies are still under investigation, all technologies and cut-off thresholds will be considered.

Target condition

The primary target condition is high-grade cervical pre-invasive disease (CIN2 or CIN3) or cervical cancer. This will be measured as CIN 2 or worse (CIN2+) for squamous cell lesions and adenocarcinoma in situ glandular lesions, as defined by histological analysis of a cervical biopsy. Where Low-grade Squamous Intraepithelial Lesion (LSIL) and HSIL terminology is used to define CIN identified by histology, a HSIL or worse (HSIL+) definition will be accepted as equivalent to CIN2 or worse (CIN2/HSIL+) (Table 1). For some studies a threshold of CIN3 or worse (CIN3+) by histology may have been used. These will be included but analysed separately.

Table 1. Summary of cytology and histology classification systems in use by NHSCSP for squamous and glandular lesions.

For peer review only

**Cytology**		**Histology**
**NHSCSP/BAC 2013**	**Bethesda 2014**	**NHSCSP 2012**
Borderline changes in squamous cells	ASC-US<br><br>ASC-H	
Low-grade dyskaryosis	LSIL	CIN1
High-grade dyskaryosis (moderate)	HSIL	CIN2
High-grade dyskaryosis (severe)	HSIL	CIN3
High-grade dyskaryosis/?invasive SCC	HSIL<br><br>SCC	SCC
Borderline changes in endocervical cells	AGC NOS	
? Glandular neoplasia	Endocervical	L-CGIN
Endocervical type	Endometrial	
Non-cervical	Glandular	
	AGC favours neoplastic	H-CGIN
	Endocervical	
	Glandular	
	Endocervical AIS	
	Adenocarcinoma	ACC
	Endocervical	
	Endometrial	
	Extrauterine	
	NOS	

Abbreviations: *ACC: adenocarcinoma; AGC NOS: atypical glandular cells, not otherwise specified; AIS: adenocarcinoma in situ ASCUS: atypical squamous cells of undetermined significance; ASC-H: atypical squamous cells cannot exclude HSIL; BAC: British association for cytopathology; CIN: cervical intra-epithelial neoplasia; L-CGIN: low grade cervical glandular intraepithelial neoplasia; H-CGIN: high grade cervical glandular intraepithelial neoplasia; LSIL: low-grade squamous intra-epithelial lesion; HSIL: high-grade squamous intra-epithelial lesion; NHSCSP: NHS cervical screening programme; SCC: squamous cell carcinoma.*

Reference Standard

Histology, performed on a cervical tissue biopsy taken at colposcopy examination, is the preferred reference standard for diagnosis of high-grade CIN (CIN2 or worse) or cervical cancer and will be

For peer review only

included as according to colposcopy guidelines in the most recent NHS Cervical Screening Programme (NHSCSP) guidelines [15]. Where the colposcopic examination is abnormal, we will assume that the histological material taken as a reference standard is fully representative of the disease status. Of note, the NHSCSP 2020 guidelines state that women with a negative hrHPV test do not need to be referred to colposcopy for biopsy. Additionally, a tissue biopsy is not clinically indicated in the presence of a normal colposcopy. Therefore, for ethical reasons, we will not require a histological biopsy for the normal grading of disease where there is either a negative hrHPV test, normal cytology or a normal colposcopy.

Types of studies

Randomised control trials, cross-sectional studies, longitudinal cohort studies, and both prospective and retrospective case-control studies, will be included where they assess the diagnostic test accuracy of human or HPV methylation marker to detect high-grade pre-invasive cervical disease as confirmed by histology.

Case reports will be excluded, as will studies in cell lines or tumour clones only. Studies that used only cytology as a reference standard will be excluded, and studies which do not use colposcopy directed histological biopsy for confirmation of a positive disease status (CIN1 or worse). We will exclude any studies where the number of participants with CIN2+ is less than 5 which are likely to give unreliable overall estimates.

Information sources and Search strategy

The literature search will start from inception and will include studies published up to the present day.

Separate search strategies will be developed for MEDLINE Ovid and Embase Ovid [25]. Searches will be conducted without language or publication status restrictions. A draft Medline strategy can be found in Appendix 1. For databases other than MEDLINE, the search strategy will be adapted accordingly. The following trial registries will be searched for ongoing studies: US National Institutes of Health Ongoing Trials Register ClinicalTrials.gov; World Health Organization International Clinical Trials Registry Platform.

We will also search the MetaRegister of Controlled Trials (mRCT), National Cancer Institute (NCI) Physisicans Data Query (PDQ), ClinicalTrials.gov and NCI Clinical Trials will be searched for ongoing studies. The main investigators of ongoing studies will be contacted for further information in case of unclear data, to determine study eligibility. Forward citation searching will be performed in Scopus/Web of Science. To identify studies which might have been missed during the electronic search,

For peer review only

the citation lists of included studies and relevant reviews will be hand-searched and experts in the field, including directors of UK cancer and colposcopy registries, contacted to identify further reports of studies. We will further check the proceedings of conferences via the Conference Proceeding Citation Index-science (CPCI-S), and unpublished eprints via MedRxiv and specifically the following relevant conferences:

- Annual Meeting of the British Society of Colposcopy and Cervical Pathology.
- Annual Meeting of the International Federation of Cervical Pathology and Colposcopy.
- EUropean Research Organisation on Genital Infection and Neoplasia (EUROGIN).
- European Federation of Colposcopy Congresses.
- Annual Meeting of the American Society of Colposcopy and Cervical Pathology.

Both published and unpublished studies will be included if they meet the inclusion criteria for the systematic review.

The review is registered with PROSPERO; the registration number is CRD42022299760.

<u>Selection process</u>

We will use reference management database EndNote [26]. Two review authors will screen all titles and abstracts. We will exclude those studies which clearly do not meet the inclusion criteria and will obtain copies of the full text of potentially relevant references. Two review authors will independently assess the eligibility of retrieved papers. We will then compare the results and resolve any disagreements by discussion. Disagreements will be resolved by consensus, with discussion with a third review author if necessary. We will document reasons for exclusion.

<u>Data extraction</u>

Two review authors will independently perform data extraction using a data extraction form. Disagreements will be resolved by consensus, with discussion with a third review author if necessary. We will extract data on the following items:

- Study design, population and setting: author, year, study design, any randomisation, study population, geography, ethnicity, total number of participants, hrHPV status (positive or negative), hrHPV assay used to define positivity, hrHPV genotype of HPV positive participants;
- Index test: technology, CpG site(s) and location, gene, hrHPV genotype for HPV tests, cut-off threshold used;
- Sample material: e.g. vaginal swab, cervical swab, endocervical curettage;

For peer review only

- Results: true positives, false positives, true negatives, false negatives will be extracted for each test and outcome (including for comparator test where available);
- Comparator: any comparator test including details of the technology;
- Reference standard: histological grade, cytological grade;
- Outcome measures e.g. CIN2+, CIN3+

We will extract data for different HPV types separately and for both individual CpG sites and combinations of CpG sites where available. Where data are missing (i.e. individual CpG sites data, or the absolute number of true- or false- positive or negative), we will contact the authors and collate additional data for studies with missing data. Where data for individual CpG sites is not provided by the author, we will perform the analysis at the gene level, or using combinations of CpG sites and present this appropriately. Our priority outcome is CIN2+, as this is currently the most clinically significant diagnosis [27].

Risk of bias in individual studies

Two authors will assess the risk of bias for eligible studies using the QUADAS-2 tool independently [28,29]. We will assign a judgement of low, moderate or high risk of bias to each of the domains (A) patient selection; B) index test; C) reference test; D) flow and timing, according to the criteria outlined in the Cochrane Handbook [30]. We will assess each domain using the signalling questions and answering Yes (Y), No (N) or Unclear (U). We will tailor the signalling questions to the review as summarised in Appendix 2. An overall judgement will be formed for risk of bias by recording information used to support the risk of bias judgement, the signalling questions and the judgement of high, low or unclear. For applicability we will record information on the applicability of each study, and rate as low, high or unclear (no score is applied for the flow and timing domain under applicability as according to QUADAS-2 guidance). For comparator studies, we will use the QUADAS-C tool to assess risk of bias [31].

We will check multiple sources to decrease the possibility of reporting and publication bias (for example, searching grey literature, citations of included studies and relevant reviews). We will assess whether only small studies (those with less than 100 participants) provide supporting evidence and will conduct a sensitivity analysis to assess robustness of results using large studies only.

Data synthesis

For peer review only

In order to define methylation positivity, we will aim to explore and summarise different cut-off points of methylation used in the literature. When several studies present duplicate data from the same population, the study with the largest sample size will be selected for inclusion in the analysis.

For absolute diagnostic accuracy, we will use the true positive (TP), true negative (TN), false negative (FN) and false positive (FP), as defined by the individual studies in terms of presence of high-grade CIN or cancer (CIN2/HSIL+) in the index test against the reference standard. Where data for the absolute sensitivity and specificity for the detection of CIN3 + or cervical cancer is provided, we will calculate this separately.

It is important to note that different HPV genotypes and genes require different test assays, and these cannot be included in the same meta-analyses. We will therefore aim to pool accuracy from tests that identify similar targets, which we anticipate will involve several different analyses:

a) Analysis of tests using the same HPV genotype and CpG sites (human or viral);

b) Analysis of tests using the same HPV genotypes and genes (individual CpG sites are not available);

c) Analysis of tests using multiple CpG sites across different genes and viral or human genomes (where the exact same combination of multiple CpG sites has been performed in different populations).

Different technologies (such as QMSP and pyrosequencing) will be combined in the above analyses, if the same CpG sites or same genes have been included.

We will first restrict to studies reporting a common threshold and will use the bivariate model [32] to estimate a summary sensitivity and specificity. Each study will contribute to the analysis once using TP, FN, TN, and FP (or 2x2 table). If there are sufficient data, we will additionally explore how different thresholds impact on the estimation of sensitivity and specificity using separate bivariate models. If there are no sufficient data to perform separate bivariate models, we will apply the hierarchical summary receiver operating curve (HSROC) model [33] to estimate a summary curve from studies that have used different test thresholds. In the case of multiple thresholds within each study, with different thresholds between studies, we will perform a linear mixed effects model accounting for heterogeneity and correlation between groups and using the TP, TN, FP, and FN values from all thresholds of each study. This model will estimate the optimal threshold, that is the point where the test is likely to perform best using the maximum value of the weighted sum of sensitivity and specificity [34]. Analyses will be performed using the *glmer* function in the R package lme4 [35] and the *metadata* Stata command [36]. When the 2x2 data are incomplete across studies and cannot be reconstructed (e.g., sensitivity and/or specificity estimates are available only), we will simplify models by assuming no correlation between

For peer review only - http://bmjopen.bmj.com/site/about/guidelines.xhtml

sensitivity and specificity and perform two univariate, random-effects, standard meta-analyses for sensitivity and specificity separately. We will use the *metaprop* function in the R package meta [37]. If few studies are available in a meta-analysis (e.g., fewer than three), and effect estimates are similar enough to be combined in a meta-analysis, then a fixed effect meta-analysis will be performed, since heterogeneity variances are expected to be poorly estimated and it is inappropriate to overfit models by estimating too many parameters from few studies [38].

In the case of any studies presenting concomitant data for methylation tests compared to alternative tests (cytology or HPV16/18 genotyping) we will examine relative accuracy. In R, for meta-analysis of diagnostic test accuracy studies, the pooled relative sensitivity and specificity of the index tests versus comparator tests will be assessed by including the test as a covariate in the bivariate model [30,39]. We will compute ratios of sensitivities and specificities across tests.

Sensitivity analyses

We plan to complete sensitivity analyses by restricting to studies free of concern for the QUADAS-2 domains based on the signalling questions we have identified as the most important. Therefore, sensitivity analyses will be performed including only those studies answering yes for each of the four domains (a-d), where:

a) Appropriate patient selection where selection was consecutive or random (QUADAS-2 item 1)

b) Where the index test used an appropriate methylation test and sample material (QUADAS-2 items 6 and 7)

c) Appropriate reference standard was used (QUADAS-2 item 8)

d) Flow and timing was appropriate (QUADAS-2 items 10 and 12)

We will perform an additional sensitivity analysis where case-control studies are excluded, and a further analysis where all types of studies except randomised control trials are excluded, in order to view their effect on the results.

Additional analyses

We will assess the between-study heterogeneity through visual inspection of forest plots (variability in sensitivity and specificity). We will also sort paired forest plots by sensitivity to explore if a reverse trend in estimates of specificities is detected. If there is variation in threshold, we will also visually inspect whether the study-specific effects lie close to the summary ROC curve. The higher the scatter

For peer review only

of the study-specific effect sizes and the larger the prediction ellipse, the higher the between-study heterogeneity. We will explore if variability can be explained by sample size, threshold effects, test variations, study characteristics, or potential effect modifiers. If there are more than 10 studies available in a meta-analysis, we will explore heterogeneity by adding covariate terms using meta-regression.

We intend to assess the influence of the following characteristics in a subgroup or meta-regression analysis: the geographical location where the study was conducted (continent; high vs low and middle income country), age groups (<30, 30-59, 60+), pre- and post-menopausal populations, the ethnicity of the study population, the technology of test used, complete versus partial verification with the reference standard used, the type of index test used (viral, human or mixed methylation; targeted genes), clinician-sample or self-sample), and commercial versus non-commercial tests.

Quality of evidence

We will perform Grading of Recommendations, Assessment, Development and Evaluations (GRADE) [40] to assess our confidence in the results our and recommendations.

Ethics and dissemination

This review does not require ethical approval.

We identified four groups of potential stakeholders (academic beneficiaries; health-related agencies and decision-makers; medical practitioners; patients and public) and specific action items to effectively target them. We will publish our review in an open access journal and will present its findings at international conferences (for example European Research Organisation on Genital Infection and Neoplasia, International Papillomavirus Conference, and European Society of Gynaecological Oncology Congress). We will make the datasets available to the wider research community. We will organise a workshop with key stakeholders. We will develop information sheets and briefings, highlighting the key findings and circulate newsletters. We will engage the press with presentations and social media interviews and we will work closely with Jo's Trust charity that plays an important role in educating patient communities.

Patient and Public involvement

A Patient and Public Involvement (PPI) meeting was conducted prior to the start of planning this meta-analysis. We discussed the current evidence surrounding methylation testing with patients and cervical charity cancer leaders, and the implications this might have on future cervical cancer screening. We discussed the need for prioritisation of early detection, whilst avoiding over treatment and anxiety

For peer review only

related to false positive diagnoses. The research question was defined with the aim of assessing the accuracy of methylation markers, as this was highlighted by our PPI meeting.

Author's contributions

SB, IK and MK conceived of the study. SB, LE and IK designed the Medline search strategy and will perform the search. SB and MK designed the study objectives and inclusion/exclusion criteria. SB, MA, and MK conceived of the approach to the QUADAS-2 tool for methodological quality assessment. SB, AAV and IK have developed the statistical methodology and analysis tools. All authors (SB, LE, AAV, IK, MP, KK, JT, EP, MA, JF, MK) have been involved in the drafting of the protocol. MK is the guarantor of the review.

Funding statement

SB received funding from Wellcome Trust 4i Clinician Scientist Fellowship (P77712) and LE received funding from Imperial Health Charity (RFPR2223_27).

Competing interests

The authors declare no conflicts of interest related to the systematic review.

References

1. Organization, W.H. Cervical cancer. Available online: https://www.who.int/health-topics/cervical-cancer#tab=tab_1 (accessed on
2. Arbyn, M.; Weiderpass, E.; Bruni, L.; De Sanjosé, S.; Saraiya, M.; Ferlay, J.; Bray, F. Estimates of incidence and mortality of cervical cancer in 2018: a worldwide analysis. *The Lancet Global Health* **2020**, *8*, e191-e203, doi:10.1016/s2214-109x(19)30482-6.
3. Bouvard, V.; Baan, R.; Straif, K.; Grosse, Y.; Secretan, B.; Ghissassi, F.E.; Benbrahim-Tallaa, L.; Guha, N.; Freeman, C.; Galichet, L.; et al. A review of human carcinogens—Part B: biological agents. *The Lancet Oncology* **2009**, *10*, 321-322, doi:10.1016/s1470-2045(09)70096-8.
4. Institute, N.N.C. HPV and Cancer. Available online: https://www.cancer.gov/about-cancer/causes-prevention/risk/infectious-agents/hpv-and-cancer (accessed on
5. Chesson, H.W.; Dunne, E.F.; Hariri, S.; Markowitz, L.E. The estimated lifetime probability of acquiring human papillomavirus in the United States. *Sexually transmitted diseases* **2014**, *41*, 660-664, doi:10.1097/OLQ.0000000000000193.
6. Shanmugasundaram, S.; You, J. Targeting Persistent Human Papillomavirus Infection. *Viruses* **2017**, *9*, 229, doi:10.3390/v9080229.
7. Arbyn, M.; Weiderpass, E.; Bruni, L.; de Sanjose, S.; Saraiya, M.; Ferlay, J.; Bray, F. Estimates of incidence and mortality of cervical cancer in 2018: a worldwide analysis. *Lancet Glob Health* **2020**, *8*, e191-e203, doi:10.1016/S2214-109X(19)30482-6.
8. Jansen, E.E.L.; Zielonke, N.; Gini, A.; Anttila, A.; Segnan, N.; Vokó, Z.; Ivanuš, U.; Mckee, M.; De Koning, H.J.; De Kok, I.M.C.M.; et al. Effect of organised cervical cancer screening on cervical cancer mortality in Europe: a systematic review. *European Journal of Cancer* **2020**, *127*, 207-223, doi:10.1016/j.ejca.2019.12.013.

For peer review only

9. Kitchener, H.C.; Castle, P.E.; Cox, J.T. Chapter 7: Achievements and limitations of cervical cytology screening. *Vaccine* **2006**, *24*, S63-S70, doi:10.1016/j.vaccine.2006.05.113.
10. Lew, J.-B.; Simms, K.T.; Smith, M.A.; Hall, M.; Kang, Y.-J.; Xu, X.M.; Caruana, M.; Velentzis, L.S.; Bessell, T.; Saville, M.; et al. Primary HPV testing versus cytology-based cervical screening in women in Australia vaccinated for HPV and unvaccinated: effectiveness and economic assessment for the National Cervical Screening Program. *The Lancet Public Health* **2017**, *2*, e96-e107, doi:10.1016/s2468-2667(17)30007-5.
11. Wright, T.C.; Stoler, M.H.; Behrens, C.M.; Sharma, A.; Sharma, K.; Apple, R. Interlaboratory variation in the performance of liquid-based cytology: Insights from the ATHENA trial. *International journal of cancer* **2014**, *134*, 1835-1843, doi:10.1002/ijc.28514.
12. Koliopoulos, G.; Nyaga, V.N.; Santesso, N.; Bryant, A.; Martin-Hirsch, P.P.L.; Mustafa, R.A.; Schünemann, H.; Paraskevaidis, E.; Arbyn, M. Cytology versus HPV testing for cervical cancer screening in the general population. *Cochrane database of systematic reviews* **2017**, *2017*, CD008587, doi:10.1002/14651858.CD008587.pub2.
13. Ronco, G.D.; Dillner, J.P.; Elfström, K.M.M.P.H.; Tunesi, S.P.; Snijders, P.J.F.P.; Arbyn, M.M.D.; Kitchener, H.P.; Segnan, N.M.D.; Gilham, C.M.; Giorgi-Rossi, P.P.; et al. Efficacy of HPV-based screening for prevention of invasive cervical cancer: follow-up of four European randomised controlled trials. *The Lancet (British edition)* **2014**, *383*, 524-532, doi:10.1016/S0140-6736(13)62218-7.
14. Arbyn, M.; Ronco, G.; Anttila, A.; Meijer, C.J.L.M.; Poljak, M.; Ogilvie, G.; Koliopoulos, G.; Naucler, P.; Sankaranarayanan, R.; Peto, J. Evidence Regarding Human Papillomavirus Testing in Secondary Prevention of Cervical Cancer. *Vaccine* **2012**, *30*, F88-F99, doi:10.1016/j.vaccine.2012.06.095.
15. England, P.H. Cervical screening: programme and colposcopy management. Available online: https://www.gov.uk/government/publications/cervical-screening-programme-and-colposcopy-management (accessed on
16. McMahon, K.W.; Karunasena, E.; Ahuja, N. The Roles of DNA Methylation in the Stages of Cancer. *The cancer journal (Sudbury, Mass.)* **2017**, *23*, 257-261, doi:10.1097/PPO.0000000000000279.
17. Kottaridi, C.; Kyrgiou, M.; Pouliakis, A.; Magkana, M.; Aga, E.; Spathis, A.; Mitra, A.; Makris, G.; Chrelias, C.; Mpakou, V.; et al. Quantitative Measurement of L1 Human Papillomavirus Type 16 Methylation for the Prediction of Preinvasive and Invasive Cervical Disease. *The Journal of Infectious Diseases* **2017**, *215*, 764-771, doi:10.1093/infdis/jiw645.
18. Bowden, S.J.; Kalliala, I.; Veroniki, A.A.; Arbyn, M.; Mitra, A.; Lathouras, K.; Mirabello, L.; Chadeau-Hyam, M.; Paraskevaidis, E.; Flanagan, J.M.; et al. The use of human papillomavirus DNA methylation in cervical intraepithelial neoplasia: A systematic review and meta-analysis. *EBioMedicine* **2019**, *50*, 246-259, doi:10.1016/j.ebiom.2019.10.053.
19. Kremer, W.; Steenbergen, R.; Heideman, D.; Kenter, G.; Meijer, C. The use of host cell DNA methylation analysis in the detection and management of women with advanced cervical intraepithelial neoplasia: a review. *BJOG: An International Journal of Obstetrics & Gynaecology* **2021**, *128*, 504-514, doi:10.1111/1471-0528.16395.
20. Cuschieri, K.; Ronco, G.; Lorincz, A.; Smith, L.; Ogilvie, G.; Mirabello, L.; Carozzi, F.; Cubie, H.; Wentzensen, N.; Snijders, P.; et al. Eurogin roadmap 2017: Triage strategies for the management of HPV-positive women in cervical screening programs. *International journal of cancer* **2018**, *143*, 735-745, doi:10.1002/ijc.31261.
21. Lorincz, A.T. Virtues and Weaknesses of DNA Methylation as a Test for Cervical Cancer Prevention. *Acta cytologica* **2016**, *60*, 501-512, doi:10.1159/000450595.
22. Kelly, H.; Benavente, Y.; Pavon, M.A.; De Sanjose, S.; Mayaud, P.; Lorincz, A.T. Performance of DNA methylation assays for detection of high-grade cervical intraepithelial neoplasia (CIN2+): a systematic review and meta-analysis. *British journal of cancer* **2019**, *121*, 954-965, doi:10.1038/s41416-019-0593-4.
23. Shamseer, L.; Moher, D.; Clarke, M.; Ghersi, D.; Liberati, A.; Petticrew, M.; Shekelle, P.; Stewart, L.A. Preferred reporting items for systematic review and meta-analysis protocols (PRISMA-P) 2015: elaboration and explanation. *BMJ* **2015**, *349*, g7647-g7647, doi:10.1136/bmj.g7647.
24. Bock, C.; Halbritter, F.; Carmona, F.J.; Tierling, S.; Datlinger, P.; Assenov, Y.; Berdasco, M.; Bergmann, A.K.; Booher, K.; Busato, F.; et al. Quantitative comparison of DNA methylation assays for biomarker development and clinical applications. **2016**.
25. de Vet, H.; Eisinga, A.; Riphagen, I.; Aertgeerts, B.; Pewsner, D. Chapter 7: Searching for Studies. In: Cochrane Handbook for Systematic Reviews of Diagnostic Test Accuracy.
26. Team, T.E. *EndNote*, EndNote 20; Clarivate: Philadelphia, PA, 2013.
27. England, P.H. Colposcopic diagnosis, treatment and follow up. Available online: https://www.gov.uk/government/publications/cervical-screening-programme-and-colposcopy-management/3-colposcopic-diagnosis-treatment-and-follow-up#CIN (accessed on

For peer review only

28. Arbyn, M.D.; Verdoodt, F.P.; Snijders, P.J.F.P.; Verhoef, V.M.J.M.D.; Suonio, E.M.D.; Dillner, L.P.; Minozzi, S.M.D.; Bellisario, C.M.; Banzi, R.P.; Zhao, F.-H.P.; et al. Accuracy of human papillomavirus testing on self-collected versus clinician-collected samples: a meta-analysis. *The lancet oncology* **2014**, *15*, 172-183, doi:10.1016/S1470-2045(13)70570-9.
29. Whiting, P.F.; Rutjes, A.W.S.; Westwood, M.E.; Mallett, S.; Deeks, J.J.; Reitsma, J.B.; Leeflang, M.M.G.; Sterne, J.A.C.; Bossuyt, P.M.M. QUADAS-2: a revised tool for the quality assessment of diagnostic accuracy studies. *Annals of internal medicine* **2011**, *155*, 529-536, doi:10.7326/0003-4819-155-8-201110180-00009.
30. JJ, D.; S, W.; C, D. Chapter 4: Guide to the contents of a
Cochrane Diagnostic Test Accuracy Protocol. JJ, D., PM, B., C, G., Eds.; The Cochrane Collaboration: Cochrane Handbook for Systematic Reviews of Diagnostic Test Accuracy Version 1.0.0 2013.
31. QUADAS-C: A Tool for Assessing Risk of Bias in Comparative Diagnostic Accuracy Studies. *Annals of Internal Medicine* **2021**, *174*, 1592-1599, doi:10.7326/m21-2234 %m 34698503.
32. Reitsma, J.B.; Glas, A.S.; Rutjes, A.W.S.; Scholten, R.J.P.M.; Bossuyt, P.M.; Zwinderman, A.H. Bivariate analysis of sensitivity and specificity produces informative summary measures in diagnostic reviews. *Journal of clinical epidemiology* **2005**, *58*, 982-990, doi:10.1016/j.jclinepi.2005.02.022.
33. Rutter, C.M.; Gatsonis, C.A. A hierarchical regression approach to meta-analysis of diagnostic test accuracy evaluations. *Statistics in medicine* **2001**, *20*, 2865-2884, doi:10.1002/sim.942.
34. Steinhauser, S.; Schumacher, M.; Rücker, G. Modelling multiple thresholds in meta-analysis of diagnostic test accuracy studies. *BMC Medical Research Methodology* **2016**, *16*, doi:10.1186/s12874-016-0196-1.
35. Bates, D.; Maechler, M.; Bolker, B.; Walker, S. Fitting Linear Mixed-Effects Models Using lme4. **2015**, *67*, 1-48, doi:doi:10.18637/jss.v067.i01.
36. Nyaga, V.N.; Arbyn, M. Metadta: a Stata command for meta-analysis and meta-regression of diagnostic test accuracy data – a tutorial. *Archives of Public Health* **2022**, *80*, doi:10.1186/s13690-021-00747-5.
37. Balduzzi, S.; Rücker, G.; Schwarzer, G. How to perform a meta-analysis with R: a practical tutorial. *Evidence Based Mental Health* **2019**, *22*, 153-160, doi:10.1136/ebmental-2019-300117.
38. Takwoingi, Y.; Guo, B.; Riley, R.D.; Deeks, J.J. Performance of methods for meta-analysis of diagnostic test accuracy with few studies or sparse data. *Statistical Methods in Medical Research* **2017**, *26*, 1896-1911, doi:10.1177/0962280215592269.
39. Takwoingi, Y.; Leeflang, M.M.G.; Deeks, J.J. Empirical evidence of the importance of comparative studies of diagnostic test accuracy. *Annals of internal medicine* **2013**, *158*, 544-554, doi:10.7326/0003-4819-158-7-201304020-00006.
40. What is GRADE? Available online: https://bestpractice.bmj.com/info/toolkit/learn-ebm/what-is-grade/ (accessed on

For peer review only

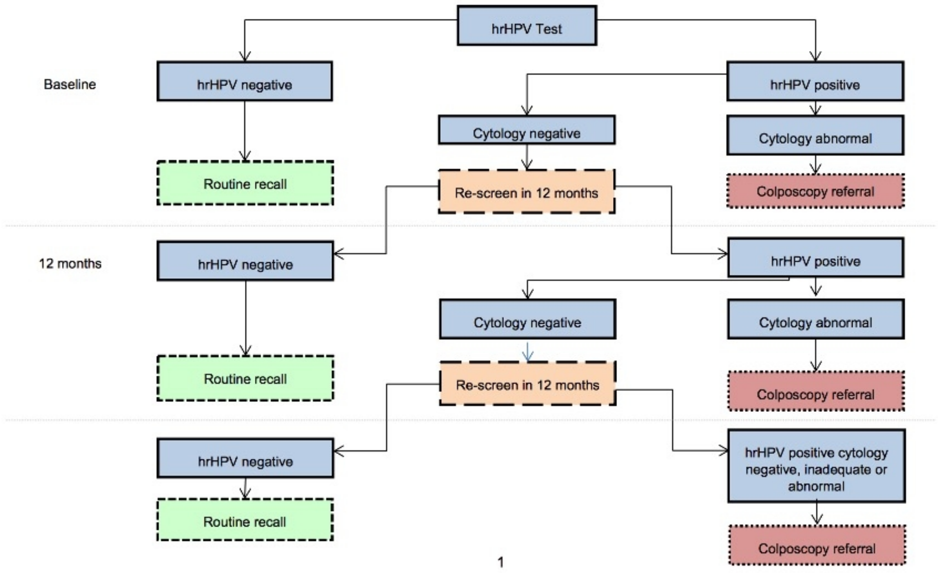

NHSCSP HPV primary screening programme. This highlights the role of the triage test (currently cytology within UK) in the cervical screening programme algorithm, for management of women detected as hrHPV positive at primary screening, and onward decision tree for colposcopy referral versus repeat screening in 12 months.

159x96mm (144 x 144 DPI)

FIGURE LEGENDS

Figure 1

NHSCSP HPV primary screening programme. This highlights the role of the triage test (currently cytology within UK) in the cervical screening programme algorithm, for management of women detected as hrHPV positive at primary screening, and onward decision tree for colposcopy referral versus repeat screening in 12 months.

Appendix 1

Search strategy

1. Uterine Cervical Neoplasms/
2. Cervical Intraepithelial Neoplasia/
3. CIN*.mp.
4. exp Uterine Cervical Dysplasia/
5. (cervi* adj5 (cancer* or tumor* or tumour* or malignan* or neoplas* or carcinoma* or adenocarcinoma* or dysplasia or squamous or CIN* or HSIL or LSIL or ASCUS)).mp.
6. 1 or 2 or 3 or 4 or 5
7. exp papillomaviridae/
8. exp Papillomavirus Infections/
9. (hpv* or papilloma*).mp.
10. 7 or 8 or 9
11. ((human or host) adj5 methylation).mp.
12. 10 or 11
13. exp methylation/
14. exp Epigenesis, Genetic/
15. Epigenomics/
16. (methylome* or epigenetic* or epigenome*).mp.
17. (bisulphite* or bisulfite* or pyrosequenc* or methylation* or mass spectrometry or mass-spectrometry or EpiTYPER).mp.
18. (next generation sequencing or NGS or high throughput sequencing or illumina array* or illumina sequenc* or solexa).mp.
19. (microsphere-based suspension array* or microsphere based suspension array* or luminex Cmap or luminex C-map or luminex C map).mp.
20. (suspension adj5 (array* or technology*)).mp.

For peer review only

21. 13 or 14 or 15 or 16 or 17 or 18 or 19 or 20
22. exp DNA/
23. DNA.mp.
24. 22 or 23
25. 21 and 24
26. exp DNA Methylation/
27. (DNA adj5 methylation*).mp.
28. 26 or 27
29. 25 or 28
30. 6 and 12 and 29

For peer review only

Appendix 2

QUADAS-2 criteria

Domain	Tailored signalling question	Description
**A) Patient Selection**		
	Was a consecutive or random sample of patients enrolled?	
	Yes	all eligible women consecutively or randomly sampled from a screening population or previous cohort
	No	non-randomly selected (e.g. cases chosen from a biobank)
	Unclear	unclear: not described
	Was a case-control design avoided	
	Yes	not case-control
	No	clear prior selection of cases and controls
	Unclear	unclear: not described
	Were inappropriate exclusions avoided?	
	Yes	yes
	No	inappropriately excluded patients
	Unclear	unclear: not described
	Applicability: Consider whether patients included differ to a hrHPV positive screening population, aged 20-70 years, have comorbid conditions not representative of screening population e.g. other cancer, HIV.	
**B) Index test**		

For peer review only

Were the index test results interpreted without knowledge of the reference standard?

Yes

No

Unclear

If a threshold was used, was it prespecified?

Yes

No

Unclear

Was there an acceptable sample material (LBC, cervical swab, cervical tissue)?

Yes	LBC, cervix swab, tissue
No	vaginal swab or other
Unclear	unclear: not described

Was there an acceptable methylation test giving quantitative results (Pyrosequencing, EpiTYPER, Next generation sequencing, Luminex)?

Yes	well established quantitative technique (Pyro, Epityper, NGS, Luminex-C)
No	binary technique - BS, MSP, MS-HRM, MSRE
Unclear	unclear

Applicability: Are there any concerns regarding the index test used and transference to a screening population of hrHPV positive women?

C) Reference Standard

Is the reference standard likely to correctly classify the target condition? (histology confirmation of grade for CIN and cancer, at least cytology confirmation of grade for normal and ASCUS)?

Yes	histology for all (CIN, Cancer) or mixed cytology and histology as clinically indicated in NHSCSP guidelines

For peer review only

No cytology for all

Unclear unclear: not described

Were the reference standard results interpreted without knowledge of the results of the index test?

Yes all scientists masked to reference test

No not masked

Unclear unclear or not described

Applicability: does the target condition in the study represent high-grade CIN or cancer?

D) Flow and timing

Was there an acceptable interval between index test and reference standard – (less than 12 weeks as the condition is unlikely to change during this time)?

Yes the index test and reference standard were performed on the same patient sample or within a reasonable time frame

No different samples used or a long delay between index test and reference standard

Unclear

Did all patients receive the same reference standard?

Yes either histology or cytology used as reference standard for all samples

No cytology as reference standard for some samples and histology used for reference standard in other samples e.g. low and high grade

Unclear unclear: not described

Were all patients included in the analysis?

Yes withdrawals explained e.g. failed methylation test

No withdrawals and uninterpretable results not explained

For peer review only

Unclear	unclear: not described

For peer review only

Reporting checklist for protocol of a systematic review and meta analysis.

Based on the PRISMA-P guidelines.

Instructions to authors

Complete this checklist by entering the page numbers from your manuscript where readers will find each of the items listed below.

Your article may not currently address all the items on the checklist. Please modify your text to include the missing information. If you are certain that an item does not apply, please write "n/a" and provide a short explanation.

Upload your completed checklist as an extra file when you submit to a journal.

In your methods section, say that you used the PRISMA-Preporting guidelines, and cite them as:

Moher D, Shamseer L, Clarke M, Ghersi D, Liberati A, Petticrew M, Shekelle P, Stewart LA. Preferred Reporting Items for Systematic Review and Meta-Analysis Protocols (PRISMA-P) 2015 statement. Syst Rev. 2015;4(1):1.

		Reporting Item	Page Number
**Title**			
Identification	#1a	Identify the report as a protocol of a systematic review	1
Update	#1b	If the protocol is for an update of a previous systematic review, identify as such	NA
**Registration**			
	#2	If registered, provide the name of the registry (such as PROSPERO) and registration number	3
**Authors**			
Contact	#3a	Provide name, institutional affiliation, e-mail address of all protocol authors; provide physical mailing address of corresponding author	1
Contribution	#3b	Describe contributions of protocol authors and identify the guarantor of the review	12

For peer review only

Amendments

	#4	If the protocol represents an amendment of a previously completed or published protocol, identify as such and list changes; otherwise, state plan for documenting important protocol amendments	NA

Support

Sources	#5a	Indicate sources of financial or other support for the review	13
Sponsor	#5b	Provide name for the review funder and / or sponsor	13
Role of sponsor or funder	#5c	Describe roles of funder(s), sponsor(s), and / or institution(s), if any, in developing the protocol	NA

Introduction

Rationale	#6	Describe the rationale for the review in the context of what is already known	3-4
Objectives	#7	Provide an explicit statement of the question(s) the review will address with reference to participants, interventions, comparators, and outcomes (PICO)	5

Methods

Eligibility criteria	#8	Specify the study characteristics (such as PICO, study design, setting, time frame) and report characteristics (such as years considered, language, publication status) to be used as criteria for eligibility for the review	6
Information sources	#9	Describe all intended information sources (such as electronic databases, contact with study authors, trial registers or other grey literature sources) with planned dates of coverage	7
Search strategy	#10	Present draft of search strategy to be used for at least one electronic database, including planned limits, such that it could be repeated	7
Study records - data management	#11a	Describe the mechanism(s) that will be used to manage records and data throughout the review	8
Study records - selection process	#11b	State the process that will be used for selecting studies (such as two independent reviewers) through each phase of the review (that is, screening, eligibility and inclusion in meta-analysis)	8

For peer review only

Study records - data collection process	#11c	Describe planned method of extracting data from reports (such as piloting forms, done independently, in duplicate), any processes for obtaining and confirming data from investigators	8
Data items	#12	List and define all variables for which data will be sought (such as PICO items, funding sources), any pre-planned data assumptions and simplifications	8
Outcomes and prioritization	#13	List and define all outcomes for which data will be sought, including prioritization of main and additional outcomes, with rationale	8-9
Risk of bias in individual studies	#14	Describe anticipated methods for assessing risk of bias of individual studies, including whether this will be done at the outcome or study level, or both; state how this information will be used in data synthesis	9
Data synthesis	#15a	Describe criteria under which study data will be quantitatively synthesised	10
Data synthesis	#15b	If data are appropriate for quantitative synthesis, describe planned summary measures, methods of handling data and methods of combining data from studies, including any planned exploration of consistency (such as I2, Kendall's τ)	10
Data synthesis	#15c	Describe any proposed additional analyses (such as sensitivity or subgroup analyses, meta-regression)	10-11
Data synthesis	#15d	If quantitative synthesis is not appropriate, describe the type of summary planned	NA
Meta-bias(es)	#16	Specify any planned assessment of meta-bias(es) (such as publication bias across studies, selective reporting within studies)	11-12
Confidence in cumulative evidence	#17	Describe how the strength of the body of evidence will be assessed (such as GRADE)	12

The PRISMA-P elaboration and explanation paper is distributed under the terms of the Creative Commons Attribution License CC-BY. This checklist was completed on 27. December 2022 using https://www.goodreports.org/, a tool made by the EQUATOR Network in collaboration with Penelope.ai

For peer review only

BMJ Open

Protocol for a Systematic Review and Meta-analysis of the Diagnostic Test Accuracy of host and HPV DNA methylation in cervical cancer screening and management

Journal:	*BMJ Open*
Manuscript ID	bmjopen-2022-071534.R1
Article Type:	Protocol
Date Submitted by the Author:	28-Apr-2023

Complete List of Authors:	Bowden, Sarah J; Imperial College London<br>Ellis, Laura; Imperial College London, Surgery and Cancer<br>Kalliala, Ilkka; Institute of Reproductive and Developmental Biology, Department of Surgery & Cancer, Faculty of Medicine, Imperial College London; University of Helsinki and Helsinki University Hospital, Department of Obstetrics and Gynaecology<br>Paraskevaidi, Maria; Imperial College London, Surgery and Cancer<br>Tighe, Jack; Imperial College London,<br>Kechagias, Konstantinos S.; Imperial College London, Surgery and Cancer<br>Doulgeraki, Triada; Ioannina University Hospital, Department of Obstetrics & Gynaecology<br>Paraskevaidis, Evangelos; University of Ioannina, Department of Obstetrics & Gynaecology<br>Arbyn, Marc ; Belgian Cancer Centre<br>Flanagan, James; Imperial College London<br>Veroniki, Areti; St Michael's Hospital Toronto<br>Kyrgiou, Maria; Imperial College London

**Primary Subject Heading**:	Obstetrics and gynaecology
Secondary Subject Heading:	Genetics and genomics
Keywords:	Gynaecological oncology < ONCOLOGY, ONCOLOGY, Colposcopy < GYNAECOLOGY, Cancer genetics < GENETICS, Gynaecological oncology < GYNAECOLOGY

SCHOLARONE™
Manuscripts

Protocol for a Systematic Review and Meta-analysis of the Diagnostic Test Accuracy of host and HPV DNA methylation in cervical cancer screening and management

Sarah J Bowden[1*], Laura Burney Ellis[1*], Ilkka Kalliala[2], Maria Paraskevaidi[2], Jack Tighe[3], Konstantinos Kechagias[2], Triada Doulgeraki[4?], Evangelos Paraskevaidis[4], Marc Arbyn[5], James Flanagan[3], Areti A Veroniki[6**], Maria Kyrgiou[7**]

[*]Joint first authors, **Joint last authors

Corresponding author = Sarah J Bowden.

s.bowden@imperial.ac.uk

laura.ellis@imperial.ac.uk

i.kalliala@imperial.ac.uk

m.paraskevaidi@imperial.ac.uk

jack.tighe18@imperial.ac.uk

konstantinos.kechagias18@imperial.ac.uk

iridoul@gmail.com

vangelispar@hotmail.com

vangelispar@hotmail.com

marc.arbyn@sciensano.be

j.flanagan@imperial.ac.uk

argie.veroniki@gmail.com

m.kyrgiou@imperial.ac.uk

[1]Surgery and Cancer, Imperial College London, London, UK
[2]The Institute of Reproductive and Developmental Biology (IRDB), Surgery and Cancer, Imperial College London, London, UK
[3] Imperial College London, London, UK
[4]Department of Obstetrics & Gynaecology, Ioannina University Hospital, Ioannina, Greece
[5]Unit of Cancer Epidemiology, Belgian Cancer Centre, Sciensano, Brussels, Belgium - Department of Human Structure and Repair, Faculty of Medicine and Health Sciences, University Ghent, Ghent, Belgium
[6]Knowledge Translation Program, Li Ka Shing Knowledge Institute, St Michael's Hospital, Unity Health Toronto, Toronto, ON, Canada - Institute of Health Policy Management and Evaluation, University of Toronto, Toronto, ON, Canada
[7]Surgery and Cancer - West London Gynaecological Cancer Centre, Imperial College London - Queen Charlotte's & Chelsea, Hammersmith Hospital, Imperial NHS Healthcare Trust, London, UK

For peer review only

Keywords

Uterine cervical neoplasms, Cervical Intraepithelial Neoplasia, DNA methylation, Epigenomics, Papillomavirus

Article Summary

Abstract

Introduction

Human papillomavirus (HPV) is necessary but not sufficient for cervical cancer development. During cervical carcinogenesis, methylation levels increase across host and HPV DNA. DNA methylation has been proposed as a test to diagnose cervical intraepithelial neoplasia (CIN); we present a protocol to evaluate the accuracy of methylation markers to detect high-grade CIN and cervical cancer.

Methods and analysis

We will search electronic databases (Medline, Embase, and Cochrane Library), from inception, to identify studies examining DNA methylation as a diagnostic marker for CIN or cervical cancer, in a cervical screening population. The primary outcome will be to assess the diagnostic test accuracy of host and HPV DNA methylation for high-grade CIN; the secondary outcomes will be to examine the accuracy of different methylation cut-off thresholds, and accuracy in high-risk HPV positive women. Our reference standard will be histology. We will perform meta-analyses using Cochrane guidelines for diagnostic test accuracy. We will use the number of true positives, false negatives, true negatives and false positives from individual studies. We will use the bivariate mixed effect model to estimate sensitivity and specificity with 95% confidence intervals; we will employ different bivariate models to estimate sensitivity and specificity at different thresholds if sufficient data per threshold. For insufficient data, the hierarchical summary receiver operating curve model will be used to calculate a summary curve across thresholds. If there is inter- and intra-study variation in thresholds, we will use a linear mixed effects model to calculate the optimum threshold. If few studies are available, we will simplify models by assuming no correlation between sensitivity and specificity and perform univariate, random-effects meta-analysis. We will assess the quality of studies using QUADAS-2 and QUADAS-C.

Ethics and dissemination

Ethical approval is not required. Results will be disseminated to academic beneficiaries, medical practitioners, patients and the public.

For peer review only

PROSPERO registration number: CRD42022299760

Strengths and Limitations of this Study

1. The results of this meta-analysis will guide targeted further research into specific genes as diagnostic markers for cervical precancer and cancer

2. We will separately analyse diagnostic test accuracy in high-risk HPV positive women, in addition to a general screening population, in order to determine the suitability of DNA methylation as a triage tool as well as a diagnostic test

3. A test with higher diagnostic test accuracy for CIN2 than cytology would have clear clinically significant impact for use as a triage or screening tool

4. Our results will be limited by the quality and quantity of studies in the review

5. Variation in measuring and reporting methylation may lead us to conduct several separate meta-analyses, rather than pooling all data

Word Count 4415

Introduction

Rationale

Cervical cancer continues to be the fourth most common cancer in women globally, resulting in 311,000 deaths in 2018 [1,2]. Human Papillomavirus (HPV) is responsible for 99% cervical cancer; about 14 genotypes of HPV are carcinogenic and classified as high-risk HPV types (hrHPV) [3,4]. Over 70% of women are infected with HPV during their lifetime [5]. In a small number, persistent hrHPV leads to development of precancerous lesions, which can progress to cancer [6].

Cervical precancer (Cervical Intraepithelial Neoplasia (CIN), classified into 3 histological grades CIN1-3), can usually be treated surgically. Where implemented, call-recall cervical screening programmes have dramatically reduced incidence of and mortality from cervical cancer through monitoring and treatment of CIN [7,8]. Such programmes have traditionally used cytology of exfoliated cervical cells as the primary screening test. Cytology testing has limitations as a screening test including a low sensitivity for CIN (53 to 75%) [9-11], high inter- and intra-observer variation and minimal future potential for robotic automation, point-of-care testing, and self-sampling. As a result, much research into alternative tests to cytology has been undertaken in recent years.

For peer review only

HrHPV DNA has been shown to be more sensitive than cytology for detection of precancerous lesions and cancer [12], and is now the primary screening method of choice in many countries, including the UK [13]. Primary hrHPV DNA screening is anticipated to offer 60 to 70% greater protection against invasive cancer compared with cytology-based screening [14]. However, higher sensitivity for CIN is associated with a decreased specificity, resulting in a modest positive predictive value [15], in part due to the fact that the hrHPV positive prevalence in countries such as the UK is as high as 13%, reaching up to 28% in women aged 30 years or less [16]. A triage test for those found to be positive for hrHPV is needed to improve the specificity of screening and identify only those who are truly at risk of precancerous and cancerous lesions.

Reflex cytology (cytology testing for hrHPV-positive samples on the same specimen), remains the test with the best historic safety data and is part of the national guidelines in many countries, including the UK [17] (Figure 1). However, molecular tests are of increasing interest, particularly human and hrHPV methylation, host methylation, HPV E6 and E7 mRNA testing and over-expression of proteins indicating progression (precancer). Such tests may offer the potential to improve accuracy in diagnosis of progressive CIN, whilst also allowing machine automation, self-sampling, and point-of-care testing in the future.

Methylation is a regulatory chemical process, which mostly occurs at specific methylation sites in DNA, called methylation 5-cytosine-phosphate-guanine-3 (CpG) sites. This causes the switching 'on and off' of specific genes to affect gene expression and determine downstream cellular processes. Methylation has been noted to be an early and frequent event in the development of many cancers, and is commonly observed at high levels in tumour tissues [18].

Studies of DNA methylation so far have focused on the variation in levels of methylation in different grades of CIN and cancer, in both human and HPV genes. Aberrant methylation levels have been noted during carcinogenesis; typically global hypomethylation, with an increase in methylation levels in tumour suppressor or tumour suppressor-like genes [19]. An increase in methylation in particular genes has been reported to be associated with increasing grades of cervical disease [20-22]. Diagnostic accuracy studies of human and HPV methylation as markers of high-grade cervical neoplasia have shown promising results [23,24], with good sensitivity and specificity [21,25]. A meta-analysis of HPV genes found HPV L1/L2 pooled sensitivity to be 77% [21], and a systematic review of methylation in host DNA found methylation of FAM19A4 to have a sensitivity between 68.2-86.7%, and specificity 60.6-79.3% [22]. Studies have reported a range of thresholds for cut-off values for DNA methylation as a diagnostic test and examined methylation .

Human and/or hrHPV methylation tests may be able to fulfil the urgent unmet need for a triage test for HPV positive women diagnosed at screening. This systematic review and meta-analysis aims to explore

For peer review only

the diagnostic test accuracy of human and/or HPV methylation tests; in women of cervical screening age, whether a positive methylation test can accurately diagnose High-grade Squamous Intraepithelial Lesion (HSIL)/CIN2+, as defined by histology as the reference standard. Our aim is to identify the best performing candidate gene markers.

Objectives

Primary Objective

The primary objective is to determine the diagnostic test accuracy of human and viral DNA methylation tests for the detection of CIN grade 2 or worse (CIN2/HSIL+), as defined by histology as a reference standard, in women of cervical screening age.

Secondary objectives

A secondary objective is to evaluate the diagnostic accuracy of human and HPV DNA methylation tests for the detection of CIN2/HSIL+, in different target populations for the use in cervical screening (where studies are available), specifically:

- Primary screening
- Triage of cytology positive women
- Triage of high-risk HPV positive women
- Post treatment of CIN

Further objectives include evaluation of the diagnostic accuracy of human and HPV DNA methylation for detection of CIN3/HSIL+, or invasive cervical cancer.

We also aim to explore the effect of different cut-off thresholds that are provided for single (or combined) methylation CpG sites (human and/or HPV) in diagnosing CIN2+ or CIN3+.

Also, in the presence of any comparator test in the same study (e.g. cytology or HPV16/18 genotyping), we will analyse relative accuracy of DNA methylation to the alternative test. Finally, in the event of sufficient data comparing self-collected and clinician-collected samples, we will analyse the relative accuracy of both methods of collection.

Methods and Analysis

This protocol was written in accordance with Preferred Reporting Items for Systematic review and Meta-Analysis Protocols (PRISMA-P) guidelines [26].

For peer review only

Patient and Public involvement

A Patient and Public Involvement (PPI) meeting was conducted prior to the start of planning this meta-analysis. We discussed the current evidence surrounding methylation testing with patients and cervical charity cancer leaders, and the implications this might have on future cervical cancer screening. We discussed the need for prioritisation of early detection, whilst avoiding over treatment and anxiety related to false positive diagnoses. The research question was defined with the aim of assessing the accuracy of methylation markers, as this was highlighted by our PPI meeting.

Eligibility criteria

Types of participants

Any population of adult women representing a cervical screening setting or referral population will be included, without demographic restrictions, including retrospective biobanks of samples for women recruited from such populations, regardless of hrHPV genotype and sample material. Where data is available, we will separately analyse hrHPV positive women.

Index tests to be considered

All DNA methylation tests will be considered: human DNA methylation tests, hrHPV DNA methylation tests or tests which combine CpG sites from human and hrHPV tests into a single marker.

Although there are several million potential CpG sites in the human genome, we will not limit the review to any particular genes and will report all findings. Within the HPV genome there are 8 genes, containing several hundred CpG sites, which vary according to HPV genotype. All genes will be considered but will be analysed separately according to HPV type. Assays can only be developed for a single genomic region spanning 2-300 base-pairs (bp) however many tests include multiple sites by combining results of different genes into one test score. We will not restrict to either single or multiple site methylation tests but will aim to analyse these separately where possible.

All human DNA and hrHPV DNA methylation technologies will be included: for example we will include (but not limited to) bisulphite clonal sequencing, pyrosequencing, epiTYPER, next generation sequencing (NGS), Luminex, Methylation-Sensitive High Resolution Melting (MS-HRM), Methylation-Specific PCR (MSP), and Illumina 450k and 850k arrays [27]. Both in-house laboratory assays and commercial assays will be included. As many technologies are still under investigation, all technologies and cut-off thresholds will be considered.

Target condition

For peer review only - http://bmjopen.bmj.com/site/about/guidelines.xhtml

The primary target condition is high-grade cervical pre-invasive disease (CIN2 or CIN3) or cervical cancer. This will be measured as CIN 2 or worse (CIN2+) for squamous cell lesions and adenocarcinoma in situ glandular lesions, as defined by histological analysis of a cervical biopsy. Where Low-grade Squamous Intraepithelial Lesion (LSIL) and HSIL terminology is used to define CIN identified by histology, a HSIL or worse (HSIL+) definition will be accepted as equivalent to CIN2 or worse (CIN2/HSIL+) (Table 1). For some studies a threshold of CIN3 or worse (CIN3+) by histology may have been used. These will be included but analysed separately.

Table 1. Summary of cytology and histology classification systems in use by NHSCSP for squamous and glandular lesions.

**Cytology**		**Histology**
**NHSCSP/BAC 2021**	**Bethesda 2014**	**NHSCSP 2012**
Borderline changes in squamous cells	ASC-US<br>ASC-H	
Low-grade dyskaryosis	LSIL	CIN1
High-grade dyskaryosis (moderate)	HSIL	CIN2
High-grade dyskaryosis (severe)	HSIL	CIN3
High-grade dyskaryosis/?invasive SCC	HSIL<br>SCC	SCC
Borderline changes in endocervical cells	AGC NOS	
?Glandular neoplasia of endocervical type	Endocervical	L-CGIN
?Glandular neoplasia (non-cervical)	Endometrial	
	Glandular	
	AGC favours neoplastic	H-CGIN
	Endocervical	
	Glandular	
	Endocervical AIS	
	Adenocarcinoma	ACC
	Endocervical	
	Endometrial	
	Extrauterine	
	NOS	

For peer review only

Abbreviations: *ACC: adenocarcinoma; AGC NOS: atypical glandular cells, not otherwise specified; AIS: adenocarcinoma in situ ASCUS: atypical squamous cells of undetermined significance; ASC-H: atypical squamous cells cannot exclude HSIL; BAC: British association for cytopathology; CIN: cervical intra-epithelial neoplasia; L-CGIN: low grade cervical glandular intraepithelial neoplasia; H-CGIN: high grade cervical glandular intraepithelial neoplasia; LSIL: low-grade squamous intra-epithelial lesion; HSIL: high-grade squamous intra-epithelial lesion; NHSCSP: NHS cervical screening programme; SCC: squamous cell carcinoma.*

Reference Standard

Histology, performed on a cervical tissue biopsy taken at colposcopy examination, is the preferred reference standard for diagnosis of high-grade CIN (CIN2 or worse) or cervical cancer and will be included as according to colposcopy guidelines in the most recent NHS Cervical Screening Programme (NHSCSP) guidelines [17,28]. Where the colposcopic examination is abnormal, we will assume that the histological material taken as a reference standard is fully representative of the disease status. Of note, the NHSCSP 2020 guidelines state that women with a negative hrHPV test do not need to be referred to colposcopy for biopsy. Additionally, a tissue biopsy is not clinically indicated in the presence of a normal colposcopy. Therefore, for ethical reasons, we will not require a histological biopsy for the normal grading of disease where there is either a negative hrHPV test, normal cytology or a normal colposcopy.

Types of studies

Randomised control trials, cross-sectional studies, longitudinal cohort studies, and both prospective and retrospective case-control studies, will be included where they assess the diagnostic test accuracy of human or HPV methylation marker to detect high-grade pre-invasive cervical disease as confirmed by histology. We will include studies assessing the accuracy of methylation markers for diagnosis only; we will not include studies assessing methylation markers as predictive markers for future development of cervical cancer or pre-invasive cervical disease.

Case reports will be excluded, as will studies in cell lines or tumour clones only. Studies that used only cytology as a reference standard will be excluded, and studies which do not use colposcopy directed histological biopsy for confirmation of a positive disease status (CIN1 or worse). We will exclude any studies where the number of participants with CIN2+ is less than 5 which are likely to give unreliable overall estimates.

Information sources and Search strategy

For peer review only

The literature search will start from inception (1946) and will include studies published up to the present day (26th April 2023).

Separate search strategies will be developed for MEDLINE Ovid and Embase Ovid [29]. Searches will be conducted without language or publication status restrictions. A draft Medline strategy can be found in Appendix 1. For databases other than MEDLINE, the search strategy will be adapted accordingly. The following trial registries will be searched for ongoing studies: US National Institutes of Health Ongoing Trials Register ClinicalTrials.gov; World Health Organization International Clinical Trials Registry Platform.

We will also search the MetaRegister of Controlled Trials (mRCT), National Cancer Institute (NCI) Physisicans Data Query (PDQ), ClinicalTrials.gov and NCI Clinical Trials will be searched for ongoing studies. The main investigators of ongoing studies will be contacted for further information in case of unclear data, to determine study eligibility. Forward citation searching will be performed in Scopus/Web of Science. To identify studies which might have been missed during the electronic search, the citation lists of included studies and relevant reviews will be hand-searched and experts in the field, including directors of UK cancer and colposcopy registries, contacted to identify further reports of studies. We will further check the proceedings of conferences via the Conference Proceeding Citation Index-science (CPCI-S), and unpublished eprints via MedRxiv and specifically the following relevant conferences:

- Annual Meeting of the British Society of Colposcopy and Cervical Pathology.
- Annual Meeting of the International Federation of Cervical Pathology and Colposcopy.
- EUropean Research Organisation on Genital Infection and Neoplasia (EUROGIN).
- European Federation of Colposcopy Congresses.
- Annual Meeting of the American Society of Colposcopy and Cervical Pathology.

Both published and unpublished studies will be included if they meet the inclusion criteria for the systematic review.

The review is registered with PROSPERO; the registration number is CRD42022299760. The registered study end date is 31st December 2023.

Selection process

We will use reference management database EndNote [30]. Two review authors will screen all titles and abstracts. We will exclude those studies which clearly do not meet the inclusion criteria and will obtain copies of the full text of potentially relevant references. Two review authors will independently

For peer review only

assess the eligibility of retrieved papers. We will then compare the results and resolve any disagreements by discussion. Disagreements will be resolved by consensus, with discussion with a third review author if necessary. We will document reasons for exclusion.

Data extraction

Two review authors will independently perform data extraction using a data extraction form. Disagreements will be resolved by consensus, with discussion with a third review author if necessary. We will extract data on the following items:

- Study design, population and setting: author, year, study design, any randomisation, study population, geography, ethnicity, total number of participants, hrHPV status (positive or negative), hrHPV assay used to define positivity, hrHPV genotype of HPV positive participants;
- Index test: technology, CpG site(s) and location, gene, hrHPV genotype for HPV tests, cut-off threshold used;
- Sample material: e.g. vaginal swab, cervical swab, endocervical curettage, urine;
- Results: true positives, false positives, true negatives, false negatives will be extracted for each test and outcome (including for comparator test where available);
- Comparator: any comparator test including details of the technology;
- Reference standard: histological grade, cytological grade;
- Outcome measures e.g. CIN2+, CIN3+, Invasive Cervical Cancer (ICC); (where individual patient-level outcome data is not available, where possible, we will contact authors to obtain this)

We will extract data for different HPV types separately and for both individual CpG sites and combinations of CpG sites where available. We will use hg38, and where studies have used hg19 or hs1, we will use LiftOver to convert these to hg38, using a LiftOver package in R [31]. Where data are missing (i.e. individual CpG sites data, or the absolute number of true- or false- positive or negative), we will contact the authors and collate additional data for studies with missing data. Where data for individual CpG sites is not provided by the author, we will perform the analysis at the gene level, or using combinations of CpG sites and present this appropriately. Our priority outcome is CIN2+, as this is currently the most clinically significant diagnosis [32].

Risk of bias in individual studies

For peer review only

Two authors will assess the risk of bias for eligible studies using the QUADAS-2 tool independently [33,34]. We will assign a judgement of low, moderate or high risk of bias to each of the domains (A) patient selection; B) index test; C) reference test; D) flow and timing, according to the criteria outlined in the Cochrane Handbook [35]. We will assess each domain using the signalling questions and answering Yes (Y), No (N) or Unclear (U). We will tailor the signalling questions to the review as summarised in Appendix 2. An overall judgement will be formed for risk of bias by recording information used to support the risk of bias judgement, the signalling questions and the judgement of high, low or unclear. For applicability we will record information on the applicability of each study, and rate as low, high or unclear (no score is applied for the flow and timing domain under applicability as according to QUADAS-2 guidance). For comparator studies, we will use the QUADAS-C tool to assess risk of bias [36].

We will check multiple sources to decrease the possibility of reporting and publication bias (for example, searching grey literature, citations of included studies and relevant reviews). We will assess whether only small studies (those with less than 100 participants) provide supporting evidence and will conduct a sensitivity analysis to assess robustness of results using large studies only (studies with 100 participants or more).

<u>Data synthesis</u>

In order to define methylation positivity, we will aim to explore and summarise different cut-off points of methylation used in the literature. When several studies present duplicate data from the same population, the study with the largest sample size will be selected for inclusion in the analysis.

For absolute diagnostic accuracy, we will use the true positive (TP), true negative (TN), false negative (FN) and false positive (FP), as defined by the individual studies in terms of presence of high-grade CIN or cancer (CIN2/HSIL+) in the index test against the reference standard. Where data for the absolute sensitivity and specificity for the detection of CIN3 + or cervical cancer is provided, we will calculate this separately.

It is important to note that different HPV genotypes and genes require different test assays, and these cannot be included in the same meta-analyses. We will therefore aim to pool accuracy from tests that identify similar targets, which we anticipate will involve several different analyses:

a) Analysis of tests using the same HPV genotype and CpG sites (human or viral);

b) Analysis of tests using the same HPV genotypes and genes (individual CpG sites are not available);

For peer review only

c) Analysis of tests using multiple CpG sites across different genes and viral or human genomes (where the exact same combination of multiple CpG sites has been performed in different populations).

Different technologies (such as QMSP and pyrosequencing) will be combined in the above analyses, if the same CpG sites or same genes have been included.

We will first restrict to studies reporting a common threshold and will use the bivariate model [37] to estimate a summary sensitivity and specificity. Each study will contribute to the analysis once using TP, FN, TN, and FP (or 2x2 table). (Where multiple studies report the same cut-off threshold in addition to other thresholds, we will choose to include data corresponding to the most commonly reported cut-off threshold. For example, we will include sensitivity and specificity from two studies evaluating methylation of the same gene, where both studies report a 10% methylation threshold.)

If there are sufficient data, we will additionally explore how different thresholds impact on the estimation of sensitivity and specificity using separate bivariate models. If there are no sufficient data to perform separate bivariate models, we will apply the hierarchical summary receiver operating curve (HSROC) model [38] to estimate a summary curve from studies that have used different test thresholds. In the case of multiple thresholds within each study, with different thresholds between studies, we will perform a linear mixed effects model accounting for heterogeneity and correlation between groups and using the TP, TN, FP, and FN values from all thresholds of each study. This model will estimate the optimal threshold, that is the point where the test is likely to perform best using the maximum value of the weighted sum of sensitivity and specificity [39]. Analyses will be performed using the *glmer* function in the R package lme4 [40] and the *metadata* Stata command [41]. When the 2x2 data are incomplete across studies and cannot be reconstructed (e.g., sensitivity and/or specificity estimates are available only), we will simplify models by assuming no correlation between sensitivity and specificity and perform two univariate, random-effects, standard meta-analyses for sensitivity and specificity separately. We will use the *metaprop* function in the R package meta [42]. If few studies are available in a meta-analysis (e.g., fewer than three), and effect estimates are similar enough to be combined in a meta-analysis, then a fixed effect meta-analysis will be performed, since heterogeneity variances are expected to be poorly estimated and it is inappropriate to overfit models by estimating too many parameters from few studies [43].

In the case of any studies presenting concomitant data for methylation tests compared to alternative tests (cytology or HPV16/18 genotyping) we will examine relative accuracy. In R, for meta-analysis of diagnostic test accuracy studies, the pooled relative sensitivity and specificity of the index tests versus comparator tests will be assessed by including the test as a covariate in the bivariate model [35,44]. We will compute ratios of sensitivities and specificities across tests. Finally, where possible, we will compute pooled positive predictive values and negative predictive values, and transform this into a pre-

For peer review only

test post-test probability plot, to facilitate the understanding of how these results can impact clinical practice.

Sensitivity analyses

We plan to complete sensitivity analyses by restricting to studies free of concern for the QUADAS-2 domains based on the signalling questions we have identified as the most important. Therefore, sensitivity analyses will be performed including only those studies answering yes for each of the four domains (a-d), where:

a) Appropriate patient selection where selection was consecutive or random (QUADAS-2 item 1)

b) Where the index test used an appropriate methylation test and sample material (QUADAS-2 items 6 and 7)

c) Appropriate reference standard was used (QUADAS-2 item 8)

d) Flow and timing was appropriate (QUADAS-2 items 10 and 12)

We will perform an additional sensitivity analysis where case-control studies are excluded, and a further analysis where all types of studies except randomised control trials are excluded, in order to view their effect on the results.

By default, studies using older technologies for the methylation test will be excluded under the QUADAS-2 domain b; in this way we will perform a sensitivity analysis for studies where only an appropriate methylation test was used. Although it will not be possible to evaluate the accuracy of technologies themselves in this review, we will use prior expert knowledge from the literature on the most accurate technologies to inform the sensitivity analyses. In this way we will explore the effect of restricting to high-quality technologies, such as pyrosequencing and next generation sequencing on diagnostic test accuracy estimates.

Additional analyses

We will assess the between-study heterogeneity through visual inspection of forest plots (variability in sensitivity and specificity). We will also sort paired forest plots by sensitivity to explore if a reverse trend in estimates of specificities is detected. If there is variation in threshold, we will also visually inspect whether the study-specific effects lie close to the summary ROC curve. The higher the scatter of the study-specific effect sizes and the larger the prediction ellipse, the higher the between-study heterogeneity. We will explore if variability can be explained by sample size, threshold effects, test

For peer review only

variations, study characteristics, or potential effect modifiers. If there are more than 10 studies available in a meta-analysis, we will explore heterogeneity by adding covariate terms using meta-regression.

We intend to assess the influence of the following characteristics in a subgroup or meta-regression analysis: the geographical location where the study was conducted (continent; high vs low and middle income country), age groups (<30, 30-59, 60+), pre- and post-menopausal populations, the ethnicity of the study population, the technology of test used, complete versus partial verification with the reference standard used, the type of index test used (viral, human or mixed methylation; targeted genes), clinician-sample or self-sample), and commercial versus non-commercial tests.

Quality of evidence

We will perform Grading of Recommendations, Assessment, Development and Evaluations (GRADE) [45] to assess our confidence in the results our and recommendations.

What this study adds

Previous meta-analyses of methylation markers as diagnostic tests have been published [25]. Whilst our study provides an update of over 4 years of data to previous studies, we also aim to address previous methodological issues with an exploration of both the generalised population of women of screening age and the specific population of HPV positive women detected at screening, with heterogeneity analyses, and sensitivity analyses to separately analyse single CpG sites. We will use a linear mixed effects model which will allow us to present the optimum threshold of both sensitivity and specificity. We will display our results in forest plots, and employ a pre-test post-test probability plot to facilitate direct potential clinical interpretation of data for cervical screening programmes. We will assess certain characteristics, including age, in sub-group meta-regression analyses. Importantly, we will perform a validated risk of bias assessment (QUADAS-2 and QUADAS-C), and perform a sensitivity analysis excluding studies with a high risk of bias. We will also quantify the certainty of the evidence using GRADE.'

Ethics and dissemination

This review does not require ethical approval.

We identified four groups of potential stakeholders (academic beneficiaries; health-related agencies and decision-makers; medical practitioners; patients and public) and specific action items to effectively target them. We will publish our review in an open access journal and will present its findings at

For peer review only

international conferences (for example European Research Organisation on Genital Infection and Neoplasia, International Papillomavirus Conference, and European Society of Gynaecological Oncology Congress). We will make the datasets available to the wider research community. We will organise a workshop with key stakeholders. We will develop information sheets and briefings, highlighting the key findings and circulate newsletters. We will engage the press with presentations and social media interviews and we will work closely with Jo's Trust charity that plays an important role in educating patient communities.

Author's contributions

SB, IK and MK conceived of the study. SB, LE and IK designed the Medline search strategy and will perform the search. SB and MK designed the study objectives and inclusion/exclusion criteria. SB, MA, and MK conceived of the approach to the QUADAS-2 tool for methodological quality assessment. SB, AAV and IK have developed the statistical methodology and analysis tools. All authors (SB, LE, AAV, IK, MP, KK, JT, EP, MA, JF, MK) have been involved in the drafting of the protocol. MK is the guarantor of the review.

Funding statement

SB received funding from Wellcome Trust 4i Clinician Scientist Fellowship (P77712) and LE received funding from Imperial Health Charity (RFPR2223_27).

Competing interests

The authors declare no conflicts of interest related to the systematic review.

FIGURE LEGENDS

Figure 1

NHSCSP HPV primary screening programme. This highlights the role of the triage test (currently cytology within UK) in the cervical screening programme algorithm, for management of women detected as hrHPV positive at primary screening, and onward decision tree for colposcopy referral versus repeat screening in 12 months.

References

For peer review only

1. Organization, W.H. Cervical cancer. Available online: https://www.who.int/health-topics/cervical-cancer#tab=tab_1 (accessed on
2. Arbyn, M.; Weiderpass, E.; Bruni, L.; De Sanjosé, S.; Saraiya, M.; Ferlay, J.; Bray, F. Estimates of incidence and mortality of cervical cancer in 2018: a worldwide analysis. *The Lancet Global Health* **2020**, *8*, e191-e203, doi:10.1016/s2214-109x(19)30482-6.
3. Bouvard, V.; Baan, R.; Straif, K.; Grosse, Y.; Secretan, B.; Ghissassi, F.E.; Benbrahim-Tallaa, L.; Guha, N.; Freeman, C.; Galichet, L.; et al. A review of human carcinogens—Part B: biological agents. *The Lancet Oncology* **2009**, *10*, 321-322, doi:10.1016/s1470-2045(09)70096-8.
4. Institute, N.N.C. HPV and Cancer. Available online: https://www.cancer.gov/about-cancer/causes-prevention/risk/infectious-agents/hpv-and-cancer (accessed on
5. Chesson, H.W.; Dunne, E.F.; Hariri, S.; Markowitz, L.E. The estimated lifetime probability of acquiring human papillomavirus in the United States. *Sexually transmitted diseases* **2014**, *41*, 660-664, doi:10.1097/OLQ.0000000000000193.
6. Shanmugasundaram, S.; You, J. Targeting Persistent Human Papillomavirus Infection. *Viruses* **2017**, *9*, 229, doi:10.3390/v9080229.
7. Arbyn, M.; Weiderpass, E.; Bruni, L.; de Sanjose, S.; Saraiya, M.; Ferlay, J.; Bray, F. Estimates of incidence and mortality of cervical cancer in 2018: a worldwide analysis. *Lancet Glob Health* **2020**, *8*, e191-e203, doi:10.1016/S2214-109X(19)30482-6.
8. Jansen, E.E.L.; Zielonke, N.; Gini, A.; Anttila, A.; Segnan, N.; Vokó, Z.; Ivanuš, U.; Mckee, M.; De Koning, H.J.; De Kok, I.M.C.M.; et al. Effect of organised cervical cancer screening on cervical cancer mortality in Europe: a systematic review. *European Journal of Cancer* **2020**, *127*, 207-223, doi:10.1016/j.ejca.2019.12.013.
9. Kitchener, H.C.; Castle, P.E.; Cox, J.T. Chapter 7: Achievements and limitations of cervical cytology screening. *Vaccine* **2006**, *24*, S63-S70, doi:10.1016/j.vaccine.2006.05.113.
10. Lew, J.-B.; Simms, K.T.; Smith, M.A.; Hall, M.; Kang, Y.-J.; Xu, X.M.; Caruana, M.; Velentzis, L.S.; Bessell, T.; Saville, M.; et al. Primary HPV testing versus cytology-based cervical screening in women in Australia vaccinated for HPV and unvaccinated: effectiveness and economic assessment for the National Cervical Screening Program. *The Lancet Public Health* **2017**, *2*, e96-e107, doi:10.1016/s2468-2667(17)30007-5.
11. Wright, T.C.; Stoler, M.H.; Behrens, C.M.; Sharma, A.; Sharma, K.; Apple, R. Interlaboratory variation in the performance of liquid-based cytology: Insights from the ATHENA trial. *International journal of cancer* **2014**, *134*, 1835-1843, doi:10.1002/ijc.28514.
12. Koliopoulos, G.; Nyaga, V.N.; Santesso, N.; Bryant, A.; Martin-Hirsch, P.P.L.; Mustafa, R.A.; Schünemann, H.; Paraskevaidis, E.; Arbyn, M. Cytology versus HPV testing for cervical cancer screening in the general population. *Cochrane database of systematic reviews* **2017**, *2017*, CD008587, doi:10.1002/14651858.CD008587.pub2.
13. England, P.H. Cervical screening: programme overview. **2021**.
14. Ronco, G.D.; Dillner, J.P.; Elfström, K.M.M.P.H.; Tunesi, S.P.; Snijders, P.J.F.P.; Arbyn, M.M.D.; Kitchener, H.P.; Segnan, N.M.D.; Gilham, C.M.; Giorgi-Rossi, P.P.; et al. Efficacy of HPV-based screening for prevention of invasive cervical cancer: follow-up of four European randomised controlled trials. *The Lancet (British edition)* **2014**, *383*, 524-532, doi:10.1016/S0140-6736(13)62218-7.
15. Arbyn, M.; Ronco, G.; Anttila, A.; Meijer, C.J.L.M.; Poljak, M.; Ogilvie, G.; Koliopoulos, G.; Naucler, P.; Sankaranarayanan, R.; Peto, J. Evidence Regarding Human Papillomavirus Testing in Secondary Prevention of Cervical Cancer. *Vaccine* **2012**, *30*, F88-F99, doi:10.1016/j.vaccine.2012.06.095.
16. Rebolj, M.; Rimmer, J.; Denton, K.; Tidy, J.; Mathews, C.; Ellis, K.; Smith, J.; Evans, C.; Giles, T.; Frew, V.; et al. Primary cervical screening with high risk human papillomavirus testing: Observational study. *BMJ (Online)* **2019**, *364*, l240-l240, doi:10.1136/bmj.l240.
17. England, P.H. Cervical screening: programme and colposcopy management. Available online: https://www.gov.uk/government/publications/cervical-screening-programme-and-colposcopy-management (accessed on
18. McMahon, K.W.; Karunasena, E.; Ahuja, N. The Roles of DNA Methylation in the Stages of Cancer. *The cancer journal (Sudbury, Mass.)* **2017**, *23*, 257-261, doi:10.1097/PPO.0000000000000279.
19. Saghafinia, S.; Mina, M.; Riggi, N.; Hanahan, D.; Ciriello, G. Pan-Cancer Landscape of Aberrant DNA Methylation across Human Tumors. *Cell Reports* **2018**, *25*, 1066-1080.e1068, doi:10.1016/j.celrep.2018.09.082.
20. Kottaridi, C.; Kyrgiou, M.; Pouliakis, A.; Magkana, M.; Aga, E.; Spathis, A.; Mitra, A.; Makris, G.; Chrelias, C.; Mpakou, V.; et al. Quantitative Measurement of L1 Human Papillomavirus Type 16 Methylation for the Prediction of Preinvasive and Invasive Cervical Disease. *The Journal of Infectious Diseases* **2017**, *215*, 764-771, doi:10.1093/infdis/jiw645.

21. Bowden, S.J.; Kalliala, I.; Veroniki, A.A.; Arbyn, M.; Mitra, A.; Lathouras, K.; Mirabello, L.; Chadeau-Hyam, M.; Paraskevaidis, E.; Flanagan, J.M.; et al. The use of human papillomavirus DNA methylation in cervical intraepithelial neoplasia: A systematic review and meta-analysis. *EBioMedicine* **2019**, *50*, 246-259, doi:10.1016/j.ebiom.2019.10.053.

22. Kremer, W.; Steenbergen, R.; Heideman, D.; Kenter, G.; Meijer, C. The use of host cell DNA methylation analysis in the detection and management of women with advanced cervical intraepithelial neoplasia: a review. *BJOG: An International Journal of Obstetrics & Gynaecology* **2021**, *128*, 504-514, doi:10.1111/1471-0528.16395.

23. Cuschieri, K.; Ronco, G.; Lorincz, A.; Smith, L.; Ogilvie, G.; Mirabello, L.; Carozzi, F.; Cubie, H.; Wentzensen, N.; Snijders, P.; et al. Eurogin roadmap 2017: Triage strategies for the management of HPV-positive women in cervical screening programs. *International journal of cancer* **2018**, *143*, 735-745, doi:10.1002/ijc.31261.

24. Lorincz, A.T. Virtues and Weaknesses of DNA Methylation as a Test for Cervical Cancer Prevention. *Acta cytologica* **2016**, *60*, 501-512, doi:10.1159/000450595.

25. Kelly, H.; Benavente, Y.; Pavon, M.A.; De Sanjose, S.; Mayaud, P.; Lorincz, A.T. Performance of DNA methylation assays for detection of high-grade cervical intraepithelial neoplasia (CIN2+): a systematic review and meta-analysis. *British journal of cancer* **2019**, *121*, 954-965, doi:10.1038/s41416-019-0593-4.

26. Shamseer, L.; Moher, D.; Clarke, M.; Ghersi, D.; Liberati, A.; Petticrew, M.; Shekelle, P.; Stewart, L.A. Preferred reporting items for systematic review and meta-analysis protocols (PRISMA-P) 2015: elaboration and explanation. *BMJ* **2015**, *349*, g7647-g7647, doi:10.1136/bmj.g7647.

27. Bock, C.; Halbritter, F.; Carmona, F.J.; Tierling, S.; Datlinger, P.; Assenov, Y.; Berdasco, M.; Bergmann, A.K.; Booher, K.; Busato, F.; et al. Quantitative comparison of DNA methylation assays for biomarker development and clinical applications. **2016**.

28. England, P.H. Cervical screening: laboratories providing HPV testing and cytology services in the NHS Cervical Screening Programme. Available online: https://www.gov.uk/government/publications/cervical-screening-laboratory-hpv-testing-and-cytology-services/cervical-screening-guidance-for-laboratories-providing-hpv-testing-and-cytology-services-in-the-nhs-cervical-screening-programme#reporting-and-classification-of-cervical-cytology (accessed on 15th January 2023).

29. de Vet, H.; Eisinga, A.; Riphagen, I.; Aertgeerts, B.; Pewsner, D. Chapter 7: Searching for Studies. In: Cochrane Handbook for Systematic Reviews of Diagnostic Test Accuracy.

30. Team, T.E. *EndNote*, EndNote 20; Clarivate: Philadelphia, PA, 2013.

31. Maintainer, B.P. *liftOver: Changing genomic coordinate systems with rtracklayer::liftOver* . **2023**.

32. England, P.H. Colposcopic diagnosis, treatment and follow up. Available online: https://www.gov.uk/government/publications/cervical-screening-programme-and-colposcopy-management/3-colposcopic-diagnosis-treatment-and-follow-up#CIN (accessed on

33. Arbyn, M.D.; Verdoodt, F.P.; Snijders, P.J.F.P.; Verhoef, V.M.J.M.D.; Suonio, E.M.D.; Dillner, L.P.; Minozzi, S.M.D.; Bellisario, C.M.; Banzi, R.P.; Zhao, F.-H.P.; et al. Accuracy of human papillomavirus testing on self-collected versus clinician-collected samples: a meta-analysis. *The lancet oncology* **2014**, *15*, 172-183, doi:10.1016/S1470-2045(13)70570-9.

34. Whiting, P.F.; Rutjes, A.W.S.; Westwood, M.E.; Mallett, S.; Deeks, J.J.; Reitsma, J.B.; Leeflang, M.M.G.; Sterne, J.A.C.; Bossuyt, P.M.M. QUADAS-2: a revised tool for the quality assessment of diagnostic accuracy studies. *Annals of internal medicine* **2011**, *155*, 529-536, doi:10.7326/0003-4819-155-8-201110180-00009.

35. Deeks, J.; Wisniewski, S.; Davenport, C. Chapter 4: Guide to the contents of a Cochrane Diagnostic Test Accuracy Protocol. *Deeks JJ, Bossuyt PM, Gatsonis C (editors), Cochrane Handbook for Systematic Reviews of Diagnostic Test Accuracy Version 1.0.0.* **2013**.

36. QUADAS-C: A Tool for Assessing Risk of Bias in Comparative Diagnostic Accuracy Studies. *Annals of Internal Medicine* **2021**, *174*, 1592-1599, doi:10.7326/m21-2234 %m 34698503.

37. Reitsma, J.B.; Glas, A.S.; Rutjes, A.W.S.; Scholten, R.J.P.M.; Bossuyt, P.M.; Zwinderman, A.H. Bivariate analysis of sensitivity and specificity produces informative summary measures in diagnostic reviews. *Journal of clinical epidemiology* **2005**, *58*, 982-990, doi:10.1016/j.jclinepi.2005.02.022.

38. Rutter, C.M.; Gatsonis, C.A. A hierarchical regression approach to meta-analysis of diagnostic test accuracy evaluations. *Statistics in medicine* **2001**, *20*, 2865-2884, doi:10.1002/sim.942.

39. Steinhauser, S.; Schumacher, M.; Rücker, G. Modelling multiple thresholds in meta-analysis of diagnostic test accuracy studies. *BMC Medical Research Methodology* **2016**, *16*, doi:10.1186/s12874-016-0196-1.

40. Bates, D.; Maechler, M.; Bolker, B.; Walker, S. Fitting Linear Mixed-Effects Models Using lme4. **2015**, *67*, 1-48, doi:doi:10.18637/jss.v067.i01.

41. Nyaga, V.N.; Arbyn, M. Metadta: a Stata command for meta-analysis and meta-regression of diagnostic test accuracy data – a tutorial. *Archives of Public Health* **2022**, *80*, doi:10.1186/s13690-021-00747-5.
42. Balduzzi, S.; Rücker, G.; Schwarzer, G. How to perform a meta-analysis with R: a practical tutorial. *Evidence Based Mental Health* **2019**, *22*, 153-160, doi:10.1136/ebmental-2019-300117.
43. Takwoingi, Y.; Guo, B.; Riley, R.D.; Deeks, J.J. Performance of methods for meta-analysis of diagnostic test accuracy with few studies or sparse data. *Statistical Methods in Medical Research* **2017**, *26*, 1896-1911, doi:10.1177/0962280215592269.
44. Takwoingi, Y.; Leeflang, M.M.G.; Deeks, J.J. Empirical evidence of the importance of comparative studies of diagnostic test accuracy. *Annals of internal medicine* **2013**, *158*, 544-554, doi:10.7326/0003-4819-158-7-201304020-00006.
45. What is GRADE? Available online: https://bestpractice.bmj.com/info/toolkit/learn-ebm/what-is-grade/ (accessed on

For peer review only

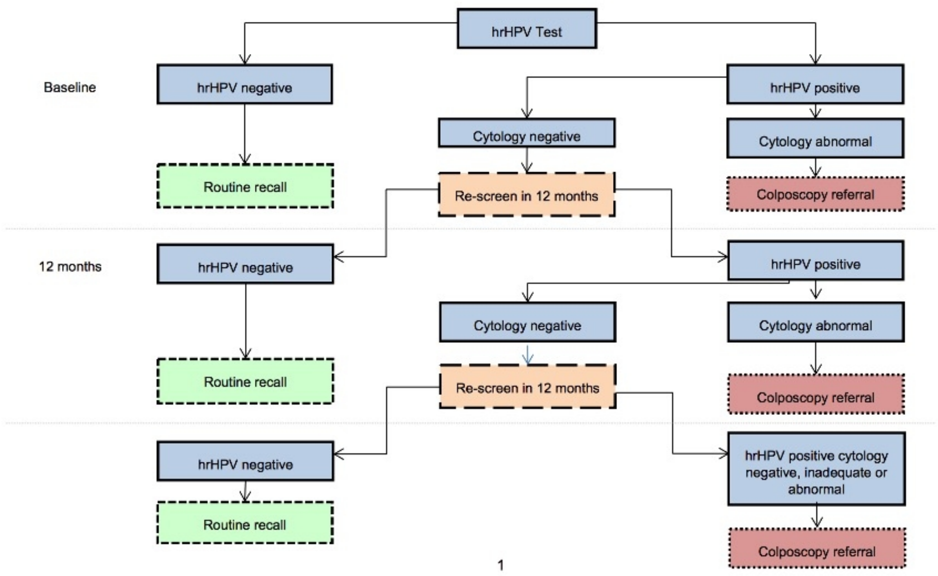

NHSCSP HPV primary screening programme. This highlights the role of the triage test (currently cytology within UK) in the cervical screening programme algorithm, for management of women detected as hrHPV positive at primary screening, and onward decision tree for colposcopy referral versus repeat screening in 12 months.

159x96mm (144 x 144 DPI)

FIGURE LEGENDS

Figure 1

NHSCSP HPV primary screening programme. This highlights the role of the triage test (currently cytology within UK) in the cervical screening programme algorithm, for management of women detected as hrHPV positive at primary screening, and onward decision tree for colposcopy referral versus repeat screening in 12 months.

Appendix 1

Search strategy

1. Uterine Cervical Neoplasms/
2. Cervical Intraepithelial Neoplasia/
3. CIN*.mp.
4. exp Uterine Cervical Dysplasia/
5. (cervi* adj5 (cancer* or tumor* or tumour* or malignan* or neoplas* or carcinoma* or adenocarcinoma* or dysplasia or squamous or CIN* or HSIL or LSIL or ASCUS)).mp.
6. 1 or 2 or 3 or 4 or 5
7. exp papillomaviridae/
8. exp Papillomavirus Infections/
9. (hpv* or papilloma*).mp.
10. 7 or 8 or 9
11. ((human or host) adj5 methylation).mp.
12. 10 or 11
13. exp methylation/
14. exp Epigenesis, Genetic/
15. Epigenomics/
16. (methylome* or epigenetic* or epigenome*).mp.
17. (bisulphite* or bisulfite* or pyrosequenc* or methylation* or mass spectrometry or mass-spectrometry or EpiTYPER).mp.
18. (next generation sequencing or NGS or high throughput sequencing or illumina array* or illumina sequenc* or solexa).mp.
19. (microsphere-based suspension array* or microsphere based suspension array* or luminex Cmap or luminex C-map or luminex C map).mp.
20. (suspension adj5 (array* or technology*)).mp.

For peer review only

21. 13 or 14 or 15 or 16 or 17 or 18 or 19 or 20
22. exp DNA/
23. DNA.mp.
24. 22 or 23
25. 21 and 24
26. exp DNA Methylation/
27. (DNA adj5 methylation*).mp.
28. 26 or 27
29. 25 or 28
30. 6 and 12 and 29

For peer review only

Appendix 2

QUADAS-2 criteria

Domain	Tailored signalling question		Description
**A) Patient Selection**			
	Was a consecutive or random sample of patients enrolled?		
	Yes	all eligible women consecutively or randomly sampled from a screening population or previous cohort	
	No	non-randomly selected (e.g. cases chosen from a biobank)	
	Unclear	unclear: not described	
	Was a case-control design avoided		
	Yes	not case-control	
	No	clear prior selection of cases and controls	
	Unclear	unclear: not described	
	Were inappropriate exclusions avoided?		
	Yes	yes	
	No	inappropriately excluded patients	
	Unclear	unclear: not described	
	Applicability: Consider whether patients included differ to a hrHPV positive screening population, aged 20-70 years, have comorbid conditions not representative of screening population e.g. other cancer, HIV.		
**B) Index test**			

For peer review only

Were the index test results interpreted without knowledge of the reference standard?

Yes

No

Unclear

If a threshold was used, was it prespecified?

Yes

No

Unclear

Was there an acceptable sample material (LBC, cervical swab, cervical tissue)?

Yes	LBC, cervix swab, tissue
No	vaginal swab or other
Unclear	unclear: not described

Was there an acceptable methylation test giving quantitative results (Pyrosequencing, EpiTYPER, Next generation sequencing, Luminex)?

Yes	well established quantitative technique (Pyro, Epityper, NGS, Luminex-C)
No	binary technique - BS, MSP, MS-HRM, MSRE
Unclear	unclear

Applicability: Are there any concerns regarding the index test used and transference to a screening population of hrHPV positive women?

C) Reference Standard

Is the reference standard likely to correctly classify the target condition? (histology confirmation of grade for CIN and cancer, at least cytology confirmation of grade for normal and ASCUS)?

Yes	histology for all (CIN, Cancer) or mixed cytology and histology as clinically indicated in NHSCSP guidelines

For peer review only

No	cytology for all
Unclear	unclear: not described

Were the reference standard results interpreted without knowledge of the results of the index test?

Yes	all scientists masked to reference test
No	not masked
Unclear	unclear or not described

Applicability: does the target condition in the study represent high-grade CIN or cancer?

D) Flow and timing

Was there an acceptable interval between index test and reference standard – (less than 12 weeks as the condition is unlikely to change during this time)?

Yes	the index test and reference standard were performed on the same patient sample or within a reasonable time frame
No	different samples used or a long delay between index test and reference standard
Unclear	

Did all patients receive the same reference standard?

Yes	either histology or cytology used as reference standard for all samples
No	cytology as reference standard for some samples and histology used for reference standard in other samples e.g. low and high grade
Unclear	unclear: not described

Were all patients included in the analysis?

Yes	withdrawals explained e.g. failed methylation test
No	withdrawals and uninterpretable results not explained

For peer review only

Unclear	unclear: not described

For peer review only

Reporting checklist for protocol of a systematic review and meta analysis.

Based on the PRISMA-P guidelines.

Instructions to authors

Complete this checklist by entering the page numbers from your manuscript where readers will find each of the items listed below.

Your article may not currently address all the items on the checklist. Please modify your text to include the missing information. If you are certain that an item does not apply, please write "n/a" and provide a short explanation.

Upload your completed checklist as an extra file when you submit to a journal.

In your methods section, say that you used the PRISMA-Preporting guidelines, and cite them as:

Moher D, Shamseer L, Clarke M, Ghersi D, Liberati A, Petticrew M, Shekelle P, Stewart LA. Preferred Reporting Items for Systematic Review and Meta-Analysis Protocols (PRISMA-P) 2015 statement. Syst Rev. 2015;4(1):1.

		Reporting Item	Page Number
**Title**			
Identification	#1a	Identify the report as a protocol of a systematic review	1
Update	#1b	If the protocol is for an update of a previous systematic review, identify as such	NA
**Registration**			
	#2	If registered, provide the name of the registry (such as PROSPERO) and registration number	3
**Authors**			
Contact	#3a	Provide name, institutional affiliation, e-mail address of all protocol authors; provide physical mailing address of corresponding author	1
Contribution	#3b	Describe contributions of protocol authors and identify the guarantor of the review	12

For peer review only

**Amendments**			
	#4	If the protocol represents an amendment of a previously completed or published protocol, identify as such and list changes; otherwise, state plan for documenting important protocol amendments	NA
**Support**			
Sources	#5a	Indicate sources of financial or other support for the review	13
Sponsor	#5b	Provide name for the review funder and / or sponsor	13
Role of sponsor or funder	#5c	Describe roles of funder(s), sponsor(s), and / or institution(s), if any, in developing the protocol	NA
**Introduction**			
Rationale	#6	Describe the rationale for the review in the context of what is already known	3-4
Objectives	#7	Provide an explicit statement of the question(s) the review will address with reference to participants, interventions, comparators, and outcomes (PICO)	5
**Methods**			
Eligibility criteria	#8	Specify the study characteristics (such as PICO, study design, setting, time frame) and report characteristics (such as years considered, language, publication status) to be used as criteria for eligibility for the review	6
Information sources	#9	Describe all intended information sources (such as electronic databases, contact with study authors, trial registers or other grey literature sources) with planned dates of coverage	7
Search strategy	#10	Present draft of search strategy to be used for at least one electronic database, including planned limits, such that it could be repeated	7
Study records - data management	#11a	Describe the mechanism(s) that will be used to manage records and data throughout the review	8
Study records - selection process	#11b	State the process that will be used for selecting studies (such as two independent reviewers) through each phase of the review (that is, screening, eligibility and inclusion in meta-analysis)	8

For peer review only

Study records - data collection process	#11c	Describe planned method of extracting data from reports (such as piloting forms, done independently, in duplicate), any processes for obtaining and confirming data from investigators	8
Data items	#12	List and define all variables for which data will be sought (such as PICO items, funding sources), any pre-planned data assumptions and simplifications	8
Outcomes and prioritization	#13	List and define all outcomes for which data will be sought, including prioritization of main and additional outcomes, with rationale	8-9
Risk of bias in individual studies	#14	Describe anticipated methods for assessing risk of bias of individual studies, including whether this will be done at the outcome or study level, or both; state how this information will be used in data synthesis	9
Data synthesis	#15a	Describe criteria under which study data will be quantitatively synthesised	10
Data synthesis	#15b	If data are appropriate for quantitative synthesis, describe planned summary measures, methods of handling data and methods of combining data from studies, including any planned exploration of consistency (such as I2, Kendall's τ)	10
Data synthesis	#15c	Describe any proposed additional analyses (such as sensitivity or subgroup analyses, meta-regression)	10-11
Data synthesis	#15d	If quantitative synthesis is not appropriate, describe the type of summary planned	NA
Meta-bias(es)	#16	Specify any planned assessment of meta-bias(es) (such as publication bias across studies, selective reporting within studies)	11-12
Confidence in cumulative evidence	#17	Describe how the strength of the body of evidence will be assessed (such as GRADE)	12

The PRISMA-P elaboration and explanation paper is distributed under the terms of the Creative Commons Attribution License CC-BY. This checklist was completed on 27. December 2022 using https://www.goodreports.org/, a tool made by the EQUATOR Network in collaboration with Penelope.ai

For peer review only
